# Microglial transcriptome analysis in the rNLS8 mouse model of TDP-43 proteinopathy reveals discrete expression profiles associated with neurodegenerative progression and recovery

**DOI:** 10.1186/s40478-021-01239-x

**Published:** 2021-08-19

**Authors:** Mandana Hunter, Krista J. Spiller, Myrna A. Dominique, Hong Xu, Francis W. Hunter, Terry C. Fang, Rebecca G. Canter, Christopher J. Roberts, Richard M. Ransohoff, John Q. Trojanowski, Virginia M.-Y. Lee

**Affiliations:** 1 Center for Neurodegenerative Disease Research (CNDR), Institute on Aging, Department of Pathology and Laboratory Medicine, Perelman School of Medicine, University of Pennsylvania, Philadelphia, PA 19104-2676 USA; 2 School of Medical Sciences, University of Auckland, Auckland, New Zealand; 3 Biogen, Cambridge, MA USA; 4 Third Rock Ventures, Boston, MA USA; 5 Present Address: Janssen Research and Development, Spring House, PA USA; 6 Present Address: Dementia Discovery Fund, SV Health Managers LLP, London, UK; 7 Present Address: Genomics Institute of the Novartis Research Foundation, San Deigo, CA USA

**Keywords:** Microglia, Amyotrophic lateral sclerosis, ALS, TDP-43, Proteinopathy, Neuroinflammation, Neurodegeneration, rNLS8, Phagocytosis, Recovery

## Abstract

The microglial reaction is a hallmark of neurodegenerative conditions, and elements thereof may exert differential effects on disease progression, either worsening or ameliorating severity. In amyotrophic lateral sclerosis (ALS), a syndrome characterized by cytoplasmic aggregation of TDP-43 protein and atrophy of motor neurons in the cortex and spinal cord, the transcriptomic signatures of microglia during disease progression are incompletely understood. Here, we performed longitudinal RNAseq analysis of cortical and spinal cord microglia from rNLS8 mice, in which doxycycline-regulatable expression of human TDP-43 (hTDP-43) in the cytoplasm of neurons recapitulates many features of ALS. Transgene suppression in rNLS8 mice leads to functional, anatomical and electrophysiological resolution that is dependent on a microglial reaction that is concurrent with recovery rather than disease onset. We identified basal differences between the gene expression profiles of microglia dependent on localization in spinal cord or cortex. Microglia subjected to chronic hTDP-43 overexpression demonstrated transcriptomic changes in both locations. We noted strong upregulation of *Apoe*, *Axl*, *Cd63*, *Clec7a*, *Csf1*, *Cst7*, *Igf1*, *Itgax*, *Lgals3*, *Lilrb4*, *Lpl* and *Spp1* during late disease and recovery. Importantly, we identified a distinct suite of differentially expressed genes associated with each phase of disease progression and recovery. Differentially expressed genes were associated with chemotaxis, phagocytosis, inflammation, and production of neuroprotective factors. These data provide new insights into the microglial reaction in TDP-43 proteinopathy. Genes differentially expressed during progression and recovery may provide insight into a unique instance in which the microglial reaction promotes functional recovery after neuronal insult.

**Supplementary Information:**

The online version contains supplementary material available at 10.1186/s40478-021-01239-x.

## Introduction

Microglia are the principal myeloid cells in the central nervous system (CNS) and are critical in maintaining the brain’s physiological microenvironment, as indicated by profound neurodegeneration which occurs with gene defects that affect microglia only among brain cells [1]. In the healthy brain, microglia comprise approximately 5–10% of CNS cells depending on location. Microglia have been implicated in amyotrophic lateral sclerosis (ALS), endoplasmic reticulum stress, mitochondrial dysfunction, loss of neurotrophic support, altered nucleocytoplasmic transport, changes in neuronal excitability and defects in axonal transport [2]. Loss of nuclear TDP-43 function may also be a key factor in the pathogenesis of ALS [3]. Nonetheless, the primary pathological events and secondary phenomena remain incompletely understood. However, the ontological association of ALS risk genes with RNA biology, protein homeostasis and axonal transport suggest these pathways to be causal [4]. Neuroinflammatory processes are also evident in ALS, with proliferation of astrocytes and microglia frequently observed [5]. The precise role of microglia in ALS is contentious [6] and may depend upon the specific molecular etiology of each presentation and on the stage of disease [7].

To examine the pathophysiology of TDP-43 proteinopathy, we previously established a transgenic mouse model with doxycycline (DOX) suppressible expression of human TDP-43 (hTDP-43) lacking a functional nuclear localization signal (∆NLS) and under the control of the neuron-specific neurofilament heavy chain (*NEFH*) promoter [8, 9]. Upon DOX withdrawal, these 'regulatable' (rNLS8) mice showed neuronal accumulation of insoluble, cytoplasmic hyperphosphorylated hTDP-43 (p-hTDP-43), leading to motor neuron loss in the hypoglossal nucleus and spinal cord, suppression of endogenous murine TDP-43 (mTDP-43), brain atrophy, muscle denervation and progressive, terminal motor impairments. Perturbation of matrix metalloproteinase-9 (MMP9), which is expressed specifically in the ALS-susceptible fast motor neurons and triggers axonal dieback via ER stress [10], preserved motor units during disease in rNLS8 mice [11]. The selective degeneration of fast motor neuron pools in the rNLS8 model recapitulated corresponding patterns observed clinically in ALS patients [12], where slow-type muscles are recalcitrant to denervation [13], supporting the physiological relevance of the model. Suppressing hTDP-43 expression in rNLS8 mice by reintroducing DOX after disease onset resolved molecular and clinical pathologies with a concurrent restoration of nuclear mTDP-43 to physiological levels, thus providing a unique model of both progression and recovery of TDP-43 proteinopathy [8].

Importantly, functional recovery in rNLS8 mice was associated with a marked expansion and morphologic changes in microglia within the spinal cord, hippocampus and cortex [14]. These reactive microglia cleared cytoplasmic hTDP-43 inclusions, while pharmacological inhibition of microgliosis via using a semi-selective CSF1R kinase inhibitor prevented functional recovery, highlighting a neuroprotective role for microglia in the rNLS8 model. Much of the incumbent understanding microglial functions in ALS is derived from murine models of familial disease, typically those driven by mutant *SOD1* in which microglia carrying the disease-causing variant actively contribute to motor neuron cytotoxicity and thus disease progression [15]. However, since the vast majority of ALS presentations are sporadic and driven by wild type TDP-43, the microglial reaction in rNLS8 mice may be more clinically relevant. Translationally, identifying the molecular mechanisms governing microglial responses in rNLS8 mice may reveal potential targets for modulating the microglial reaction in a fashion that promotes tissue repair.

In this study, we performed RNAseq analysis of primary microglia isolated from the cortex and spinal cord of rNLS mice across the time course of the disease model, including samples taken at baseline, early disease, late disease and recovery. We characterize the stage-specific transcriptomic response of microglia and identify a suite of differentially expressed genes associated with each phase of disease progression and recovery. The set of genes differentially expressed upon TDP-43 insult is characteristic of temporal regulation of interferon, chemokine, and mitogenic signalling, chemotaxis and phagocytosis.

## Materials and methods

### rNLS8 mice

The generation and husbandry of Tg(NEFH-tTA)8Vle and Tg(tetO-hTDP-43-ΔNLS)4Vle mice (#025,397, Jackson Laboratory), which are crossed to yield hemizygous rNLS8 mice, has been previously reported [8]. In these lines, administration of diet containing DOX (200 mg.kg^−1^, #S3888, Bio-Serv) sequesters the TetR domain of tTA, thereby suppressing VP16-mediated transcription at the TetO sequence linked to hTDP-43. Neuronal expression of hTDP-43 was activated by substituting DOX chow for standard diet (Rodent Diet 20 #5053, PicoLab). Male and female mice were weaned on DOX, genotyped at age 2–5 months as described [8] and rNLS8 bigenic mice were randomized to DOX treatment groups in the study. The animal cohort has been reported previously [14] and group sizes were as follows: control (on DOX to endpoint) *n* = 14 (8 females, 6 males), early disease (two weeks off DOX) *n* = 8 (5 females, 3 males), late disease (6 weeks off DOX) *n* = 7 (4 females, 3 males) and recovery (6 weeks off DOX followed by one week back on DOX) *n* = 9 (5 females, 4 males). The selection of these timepoints was informed by our previous characterization of the disease course in rNLS8 [8, 12, 14]. Early disease corresponds to the achievement of apical hTDP-43 expression, the initial presentation of motor dysfunction, presence of hTDP-43 inclusions and loss of endogenous nuclear mTDP-43 expression. Late disease is the latest disease timepoint at which all rNLS8 mice are expected to remain living, and is characterized by peak motor neuron dieback, tibialis anterior and gastrocnemius denervation, muscle atrophy, severe motor dysfunction and body weight loss. Recovery corresponds to the peak microglial density and morphological reaction, clearance of hTDP-43 inclusions and co-localization of hTDP-43 with Iba-1 staining, arresting of motor neuron dieback and initial resolution of motor dysfunction and body weight loss. Animals were handled in accordance with the US National Research Council's Guide for the Care and Use of Laboratory Animals, the US Public Health Service's Policy on Humane Care and Use of Laboratory Animals, and Guide for the Care and Use of Laboratory Animals. Studies were approved by the Institutional Animal Care and Use Committee of the University of Pennsylvania.

### Isolation of microglia

The method used for isolation of microglia from the spinal cord and cortex of rNLS8 mice has been reported [14]. Briefly, cortical and spinal cord tissues were homogenized to single-cell suspensions using Wheaton tissue grinders and centrifuged through a 30% Percoll gradient (GE Healthcare). The resulting suspensions, enriched for microglia, were Fc blocked using an anti-CD16/32 antibody (clone 93, Biolegend) and stained with CD11b-PE (clone M1/70, BD Biosciences) and CD45-BV421 (clone 30-F11, Biolegend) antibodies. DRAQ7 staining was used for exclusion of dead cells, and sorting of CD11b^+^/CD45^lo^ microglia directly in RNeasy Lysis Buffer (Qiagen) was performed using a FACSAria Fusion flow cytometer (BD). Animals were sacrificed and microglia isolated for analysis in a batch-wise manner, in which each batch contained animals from multiple treatment groups to mitigate the risk of technical variance.

### RNA sequencing

Total RNA isolation from purified microglia was carried out as reported [14]. cDNA was synthesized using a SMART-Seq v4 Ultra Low Input RNA Kit (Takara Bio) and indexed libraries prepared using a Nextera XT DNA Library Preparation Kit (Illumina). Sequencing was performed on a HiSeq 2500 (Illumina) with an average depth of ca. 30 million aligned paired-end fragments per library. Reads were trimmed using Trim Galore! v0.6.4, aligned to the *mm10* assembly using STAR v2.7 [16] and FPKM estimated using RSEM v1.3.1 [17]. RNAseq data are available from the NCBI Sequence Read Archive (accession number PRJNA624791).

### Bioinformatic analyses

**Fig. 1:**
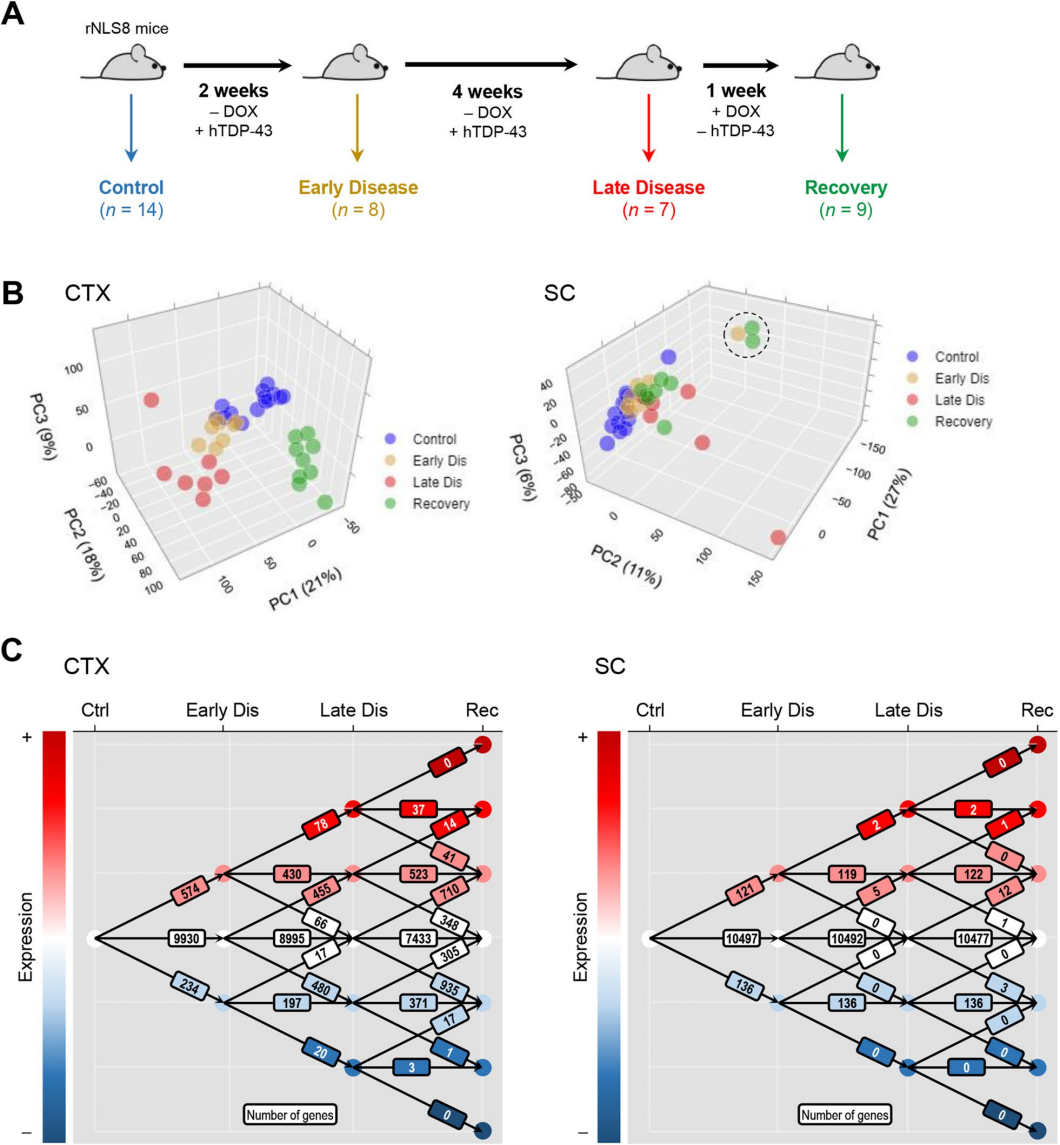
Longitudinal gene expression changes throughout disease progression and recovery in rNLS8 mice. **A** Study schema. **B** Principal component analysis of transcriptomic data (as quantile-normalized, log_2_-transformed FPKM) obtained for microglia isolated from the cortex (left panel) or spinal cord (right panel) of control mice or animals in early disease, late disease and recovery phases. Scores for the first three principal components are represented in three-dimensional scatter plots, where data points correspond to microglial isolates from individual animals and the fraction of total variance explained by each principal component is denoted in axis labels. Principal component analysis used unit variance scaling and singular value decomposition with imputation. The three circled datapoints are outlying samples excluded from the principal component analysis shown in Additional file 1: Figure S2, though these samples were not excluded from any other analyses in this study. **C** Longitudinal topography of differentially expressed genes (Benjamini–Hochberg adjusted *P* < 0.05; log_2_-fold change > 0.5) in microglia isolated from the cortex (left panel) or spinal cord (right panel) of animals over the course of disease progression and recovery. The number of genes showing increased expression (upward vectors), unchanged expression (lateral vectors), and decreased expression (downward vectors) at each disease stage transition is illustrated

Bioinformatic analyses were performed in R version 3.5.3. Transcripts for which < 1 aligned fragment was detected were censored from the dataset. FPKM values were then quantile-normalized and log_2_-transformed for subsequent analyses. Differential expression analyses used the limma method [18]. For the majority of analyses, differential expression contrasts were made relative to control animals. To define the longitudinal gene expression “topology” characterized by the set of gene expression changes occurring at each disease phase relative to the preceding phase (Fig. 1C), the differential expression contrasts ‘control vs. early disease’, ‘early disease vs. late disease’ and ‘late disease vs. recovery’ were executed. Similarly, to specifically isolate gene expression changes occurring concurrently with the transition from late disease to recovery (Fig. 6), the latter differential expression contrast was used. Transcripts that yielded adjusted P values < 0.05 after Benjamini–Hochberg correction were considered significant, with further filtering for absolute log_2_-fold change > 0.5 or > 1.0 applied as indicated in figure legends. Unsupervised hierarchical clustering used the ward.D method with Euclidean distance. Principal component analyses used unit variance scaling and singular value decomposition with imputation. Protein–protein interaction networks were generated using the STRING database [19], where the number of expected edges in a given network was estimated from randomly sampled networks of the same order. Low P-values for interactions in the network thus implied that the observed nodes were nonrandomly sampled. Enrichment of gene ontology and Reactome pathway terms among differentially expressed gene lists were computed using the PANTHER database [20]. Enrichment of gene ontology and pathway annotations among differentially expressed genes were computed relative to the sampling space of all transcripts (n = 10,773) that were detected after normalization in at least one sample in the study.

### Immunofluorescence staining

rNLS8 and non-transgenic littermate mice were perfused with ice-cold PBS followed by 10% formalin, and then the extracted brain and spinal cord were post-fixed in 10% formalin overnight. Samples were then washed in PBS and then processed in a sucrose gradient up to 30% for cryoprotective embedding. Coronal cortical and lumbar spinal cord sections were obtained by cryosectioning at 20 µm, air-dried overnight and processed for staining. Antigen retrieval was performed by heating the sections to 80 °C for 3 min in citrate buffer. Blocking was performed for 1 h incubation in PBS containing 0.3% Triton-X 100, 0.1% Tween-20, 5% donkey serum and 2% IgG-free BSA. Sections were incubated with primary antibody anti-Axl (R&D AF854, 1:50 dilution) and anti-Iba1 (Wako 019–19,741, 1:250 dilution) diluted in blocking buffer at 4 °C overnight. Sections were washed in PBS 0.1% Tween-20 and incubated for 2 h at 22–24 °C with fluorophore-coupled (Alexa Fluor 488 or 568) secondary antibodies (1:1,000, Molecular Probes) and sealed with VECTASHIELD Antifade Mounting Medium with DAPI (Vector Laboratories). Images were obtained using a Leica TCS SPE.

### In situ hybridization and immunohistochemistry

rNLS8 mice were perfused as described above, the brain removed and snap frozen on dry ice. Cortical tissue was sectioned at 7 µm thickness using a Leica CM3050 cryostat. *Axl* (ACD 450,938) or *Igf1* (ACD 443,908) probes were used in combination with anti-hTDP43 (CNDR, clone 5104; used at 0.51 µg/mL) or anti-Iba1 (Wako 019–19,741; used 1:1,000) antibodies for double labelling. Slides were first brought to room temperature then fixed in Neutral Buffered Formalin (Fisher Scientific, SF100-20) for 60 min followed by 5 min each in 50% ethanol, 70% ethanol, then 100% ethanol before air drying. Slides were stained on a Bond Rx Autostainer (Leica) using the RNAscope LSx Red staining kit (ACD, 322,750) and the Bond Polymer Refine Detection Kit (Leica DS9800). Chromogenic RNAscope was performed first using the standard protocol. Antigen retrieval was performed with E2 (Leica) at 95 °C for 20 min and protease digestion (ACD) at 40 °C for 30 min. Immunohistochemistry was then performed using the standard protocol ‘F’ with the exception of the primary antibody incubation time being extended to 60 min and the peroxide blocking step excluded. An additional rodent blocking step (Biocare Medical RBM961L) at room temperature for 20 min was included. Imaging was performed using a Nikon Eclipse Ni microscope.

## Results

### Transcriptomic analysis of cortical and spinal cord microglia from control rNLS8 mice reveal different basal levels of inflammation

We previously reported a significant numerical increase in Iba-1^+^ microglia in the lumbar spinal cord, cortex and hippocampus of rNLS8 mice following DOX-mediated suppression of the hTDP-43 transgene, which elicited microglia-dependent functional recovery [14]. This reaction was specific to the recovery phase, with only subtle changes observed in microglial density and morphology in the spinal cord during disease. To explore the molecular changes that underlie the microglial population dynamics and resultant functional recovery observed in rNLS8 mice, we performed RNA sequencing on microglia isolated from the cortex and spinal cord of animals at baseline (control, *n* = 14), early disease (*n* = 8), late disease (*n* = 7) and recovery (*n* = 9; Fig. 1A). Sporadic ALS is sexually dimorphic, with up to twice as many affected males than females [21]. Moreover, sex-specific gene expression features have been described in the microglia of C57BL/6 mice [22]. Therefore, we investigated sex as a potentially confounding variable in our expression profiles by performing principal component analysis (PCA) of normalized gene expression measures derived from cortical and spinal cord samples from male and female control mice (Additional file 1: Figure S1A). When assessed in terms of the first three principal components, which explained 20%, 10% and 8% of the total variance in the dataset, respectively, microglial gene expression profiles derived from female and male control mice were indistinguishable. The remaining 34 principal components (which collectively explained all variance in the dataset) similarly did not discriminate between sexes (Additional file 2: File 1). Formal differential expression contrasts revealed only the Y-linked genes *Eif2s3y*, *Ddx3y*, *Uty* and *Kdm5d*, in addition to *Camp* (located on chromosome 9) to be more highly expressed in male than female control cortical microglia, whereas *Selenow* was higher in females (not shown). No genes were sexually dimorphic in microglia isolated from the spinal cords of control mice. We similarly did not observe sexual dimorphism in the gene expression profiles of microglia isolated at early or late disease or at the recovery phase (not shown). Our data were thus in agreement with the general observation that microglial sexual dimorphism is strictly context- and age-dependent.

Control samples isolated from the cortex and spinal cord clustered into discrete groups, indicating phenotypic differences in microglia residing in these anatomic sites despite the nominal suppression of the hTDP-43 transgene in the control animals. Differential expression analysis revealed sets of genes associated with microglia from either the cortex or spinal cord (Additional file 1: Figure S1B, Additional file 3: File 2), which included higher expression of *Clec7a* and *Spp1* in the latter. Spinal cord microglia were more inflammatory in their transcriptomic features and genes exhibiting higher expression in these cells relative to cortical microglia encompassed a protein–protein interaction network associated with immunological and cytokine responses (Additional file 1: Figure S1C). These data identified microglia in the spinal cord of rNLS8 mice to display a more inflammatory phenotype than those in the cortex even in the absence of hTDP-43 induction.

### Longitudinal transcriptomic analysis of cortical and spinal cord microglia from rNLS8 mice after the induction of TDP-43 proteinopathy

We next examined global gene expression changes in rNLS8 cortical and spinal cord microglia temporally as a function of hTDP-43-driven disease progression and microglia-dependent recovery. In the cortex, PCA demarcated gene expression profiles from microglia isolated from control, early disease, late disease and recovery animals (Fig. 1B). Within the context of the geometric space defined by the first three principal components, a progressive gene expression shift from control was observed in early disease and then more strongly in late disease, while recovery-phase microglia displayed an expression pattern orthogonal (and thus distinct) to the prior three cohorts. Global disease-group expression profiles were less clearly delineated in the spinal cord (Fig. 1B), with three outlying samples (two recovery-phase and one early disease, all animals that were sacrificed and experimentally processed on the same day) driving variance in terms of the first principal component (Fig. 1B, circled outliers). Excluding these outliers from the PCA improved group delineation, though significant intra-group variation was still observed, particularly within control and late-disease microglial isolates (Additional file 1: Figure S2). We next defined the sets of genes showing longitudinal changes in global gene expression profiles in rNLS8 cortical and spinal cord microglia throughout disease onset, progression and recovery by computing differential expression analysis for each disease stage relative to the prior stage, thus establishing the ‘topology’ of each gene in the RNAseq dataset for subsequent analyses (Fig. 1C, Additional file 4: File 3).

### A novel set of microglial genes in rNLS8 mice discriminates disease from recovery

Prior transcriptomic studies have reported ‘disease-associated’ microglia (DAM), a putative subset of microglia that show conserved changes in their expression of a core suite of ‘DAM genes’ (Additional file 1: Table S1) in response neurodegenerative insults across multiple disease models [23, 24]. In rNLS8 mice, expression of the microglial homeostatic or surveillance genes *Cx3cr1*, *Tmem119*, *P2ry12* and *P2ry13* persisted at early disease, with increased expression among the DAM signature of only *Lilrb4*, *Cst7*, *Lpl*, *Lgals3*, *Cd63*, *Axl* and *Clec7a* evident in cortical isolates, suggesting initial microglial activation (Additional file 1: Figure S3A). No DAM genes were differentially expressed at early disease in the spinal cord. Differential expression of several DAM signature genes was evident in rNLS8 mice at late disease in both the cortex (Additional file 1: Figure S3A) and the spinal cord (Additional file 1: Figure S3B), at which time *Apoe*, *Axl*, *B2m*, *Cd63*, *Cd9*, *Clec7a*, *Csf1*, *Cst7*, *Ctsb*, *Ctsl*, *Fth1*, *Gnas*, *Igf1*, *Itgax* (Cd11c), *Lgals3*, *Lilrb4*, *Lpl*, *Lynz2* and *Spp1* were upregulated, with concomitant downregulation of microglial homeostatic markers *Cx3cr1*, *P2ry12*, *P2ry13*, *Timp2* and *Tmem119*. Notably, *Trem2* did not show elevated expression at any disease phase in RNAseq analysis of bulk microglia from rNLS8 mice.

**Fig. 2:**
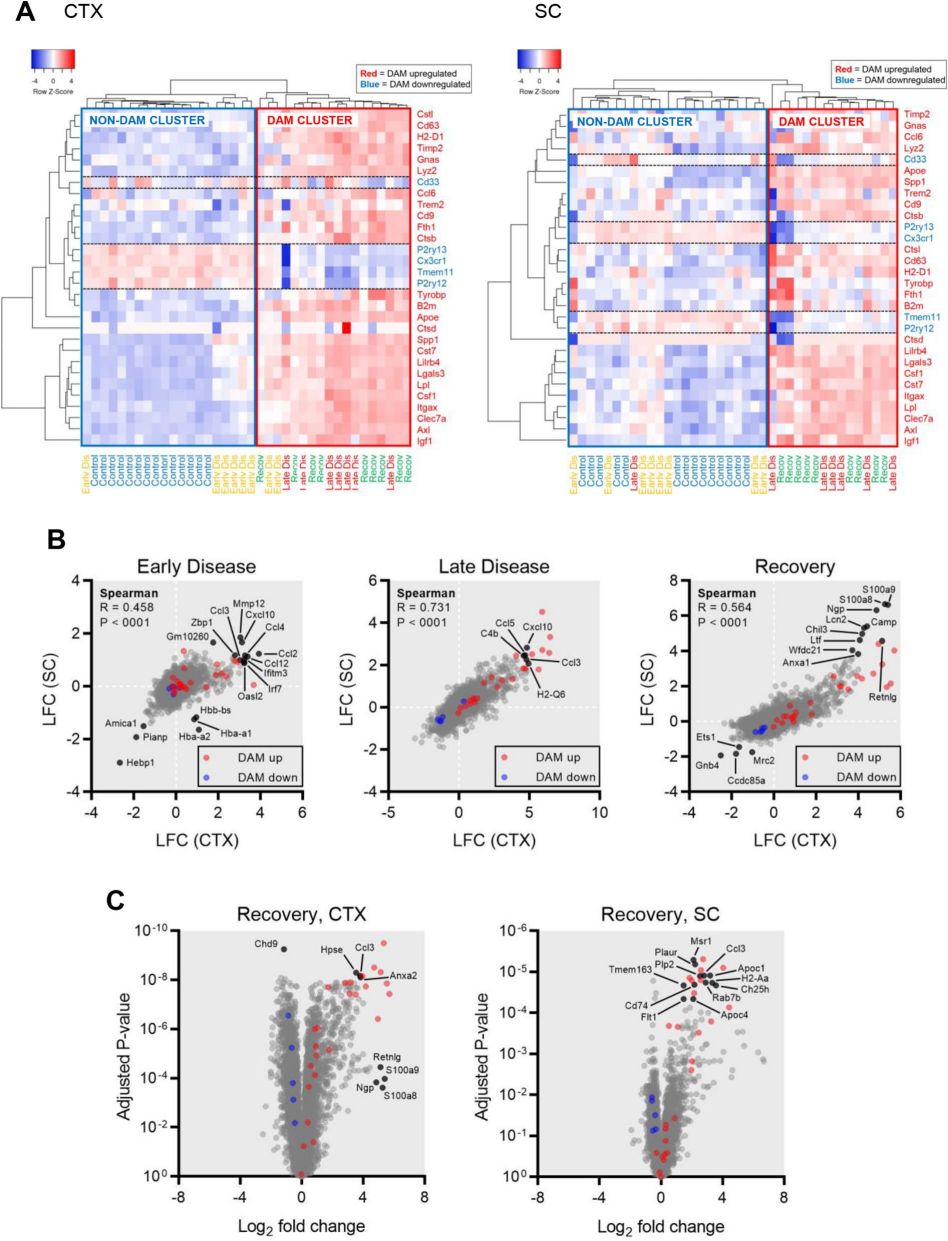
Altered expression of genes not in the DAM signature in microglia isolated from rNLS8 mice. **A** Unsupervised hierarchical clustering of microglia isolates from the cortex (left panel) or spinal cord (right panel) according to the expression values (normalized to row Z-scores as depicted in the heatmap scales) of canonical DAM genes. The reported direction of expression changes under neurodegenerative conditions is represented by the color coding of row labels. Two distinct sample clusters, a ‘non-DAM cluster’ containing all control and most early disease samples, and the ‘DAM cluster’ containing all recovery and most late disease isolates, are demarcated in the heatmaps. Clustering used the ward.D method with Euclidean distance. **B** Scatter plots of log_2_ fold change values derived from the comparison of early disease (left panel), late disease (middle panel) and recovery microglia (right panel) to control microglia in the cortex and spinal cord. Spearman correlation metrics are denoted as an assessment of the consistency of microglial gene expression changes between these two anatomical sites. Canonical DAM genes reported to be upregulated under neurodegenerative conditions are marked in red, whereas canonically downregulated DAM genes are marked in blue. **C** Volcano plots illustrating the magnitude (as log_2_ fold change) and statistical significance (as Benjamini–Hochberg adjusted *P*-values) of changes in the expression of individual genes in microglia at recovery relative to control microglia isolated from the cortex (left panel) or spinal cord (right panel). Canonical DAM genes reported to be upregulated under neurodegenerative conditions are marked in red, whereas canonically downregulated DAM genes are marked in blue

Analysis of the DAM transcriptome genes did not differentiate disease from recovery as unsupervised hierarchical clustering by DAM gene expression values demarcated two clusters – the ‘DAM cluster’ and ‘non-DAM cluster’ – in which late disease and recovery samples were both assigned to the DAM cluster and control and most early disease isolates assigned to the non-DAM cluster (Fig. 2A). In prosecuting expression analyses in rNLS8 mice, we also identified additional genes (i.e. transcripts not included in the canonical DAM gene set) that showed highly significant differential expression across disease phases. Thus, to more fully understand microglial transcriptomic phenotypes in this model, we curated an expanded set of genes that showed altered expression in rNLS8 microglia throughout disease progression and recovery. The observed gene expression changes were broadly correlated (in terms of gene-wise log_2_-fold change values relative to control animals) between cortical and spinal cord isolates at each disease phase, indicating consistent microglial responses to hTDP-43-mediated pathology (Fig. 2B). This observation was consistent with the fact that the time-dependence and extent of neuronal injury in the brain and spinal cord is equivalent in rNLS8 mice [8]. We noted larger expression log-fold changes in cortical than in spinal cord samples, potentially reflecting the ostensibly higher level of basal (control) inflammation and great inter-sample technical variance at the latter site. At early disease, *Mmp12*, *Cxcl10*, *Ccl2*, *Ccl12*, *Zbp1*, *Gm10260*, *Ifitm3*, and *Oasl2* were among the genes showing greatest expression increases, while *Amica1*, *Pianp* and *Hebp1* showed decreased expression, with *Hbb-bs*, *Hba-a1* and *Hba-a2* demonstrating decreased expression in spinal cord but not cortical microglia. The expression of *Cxcl10*, *Ccl5*, *C4b*, *Ccl3*, and *H2-Q6* was particularly high in late disease. A distinct set of genes were modulated in recovery-phase microglia, where *S100a8*, *S100a9* (collectively encoding calprotectin), *Ngp*, *Camp*, *Lcn2*, *Chil3*, *Ltf*, *Wfdc1*, *Anxaa*, *Retnlg*, *Msr1*, *Plaur*, *Plp2*, *Tmem163*, *Cd74*, *Flt1*, *Apoc4*, *Rab7b*, *Ch25h*, *H2-Aa*, *Apoc1*, and *Hpse* all exhibited increased expression, while the expression of *Ets1*, *Mrc2*, *Gnb4*, *Ccdc85a* and *Chd9* was decreased (Fig. 2B-C). Genes showing elevated expression in early and late disease microglia in the cortex (Additional file 1: Figure S4) and spinal cord (Additional file 1: Figure S5) were enriched for gene ontology (GO, biological process sub-category) and Reactome pathway annotations relating to immune responses and effector processes, IFN, chemokine, cytokine and TNF signalling. We also identified a gene signature associated with ERK1 and ERK2 signalling (a subset of GO:0,070,372, ‘regulation of ERK1 and ERK2 cascade’) that was elevated in late disease and recovery (Additional file 1:Figure S6) and potentially associated with microglial proliferation. To explore the potential drivers of this tyrosine kinase cascade signature, we examined the expression of key microglial receptors (*Axl*, *Mertk*, *Csf1r*, *Igf1r*, *Tlr4*, *Flt1*) and corresponding ligands (*Gas6*, *Pros1*, *Csf1*, *Igf1*; Additional file 1: Figure S7). Among these, *Axl*, *Csf1*, *Igf1* and *Flt1* showed elevated expression concurrent with the ERK1/ERK2 signature, suggesting that microglial proliferation in rNLS8 mice may be regulated via these axes.

**Fig. 3:**
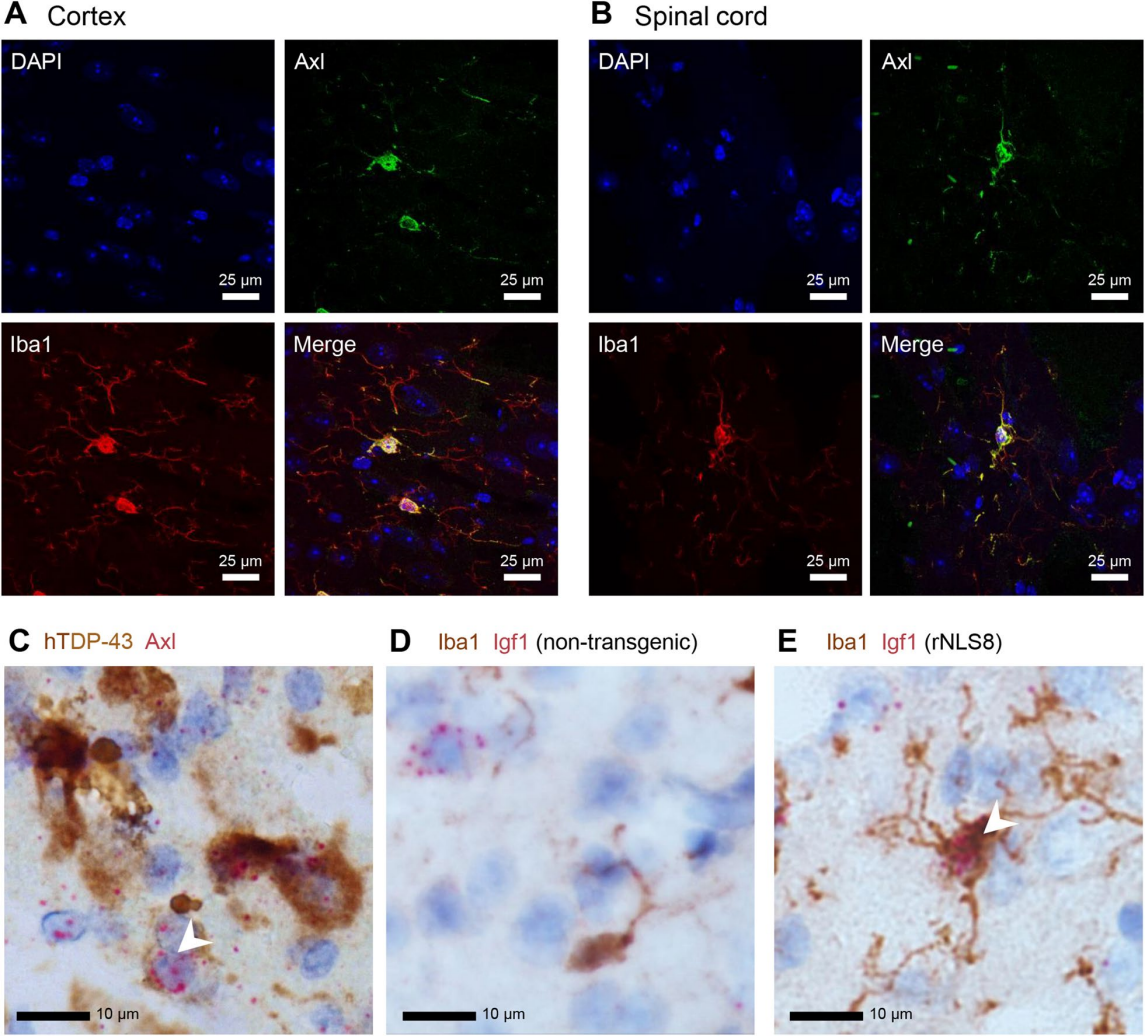
In situ protein and mRNA expression analysis for Axl and Igf1 in Iba1-positive microglia. Immunofluorescence imaging for Axl and Iba1 in representative cortical **A** and spinal cord **B** sections from rNLS8 mice at the late disease stage (i.e., DOX withdrawn for 6 weeks). **C** In situ hybridization for *Axl* mRNA (red) with hTDP-43 immunohistochemical co-staining (brown) in an rNLS8 cortical section, showing Axl-expressing microglia (white arrow) proximal to sites of hTDP-43 deposition. In situ hybridization for *Igf1* mRNA (red) with Iba1 immunohistochemical co-staining (brown) in cortical sections from wild type/non-transgenic **D** and rNLS8 mice **E**, where the white arrow marks *Axl*-expressing Iba1-positive microglia

Since *Axl* and *Igf1* were two of the most strongly upregulated genes during the disease phase and have both been implicated in microglial reactions to neuropathological insult [25, 26], we performed in situ imaging studies to validate specific expression in rNLS8 microglia. While no expression of Axl protein was detectable in Iba1-positive microglia by immunofluorescence staining of cortical and spinal cord sections from non-transgenic littermates (Additional file 1: Figure S8), Axl was expressed and co-localized with Iba1 in ramified microglia in the cortex and spinal cord of rNLS8 mice at late disease (Fig. 3A-B). Using RNAscope in situ hybridization, we observed *Axl*-positive nuclei (presumed microglia) proximal to sites of hTDP-43 protein accumulation detected by immunohistochemical co-labelling at late disease (Fig. 3C). Similarly, we did not detect *Igf1* mRNA in Iba1-positive microglia in cortical sections from non-transgenic littermates (though expression was seen in Iba1-negative cells; Fig. 3D). In contrast, *Igf1* transcripts were detected in Iba1-positive microglia in cortical sections from rNLS8 mice at late disease (Fig. 3E).

**Fig. 4:**
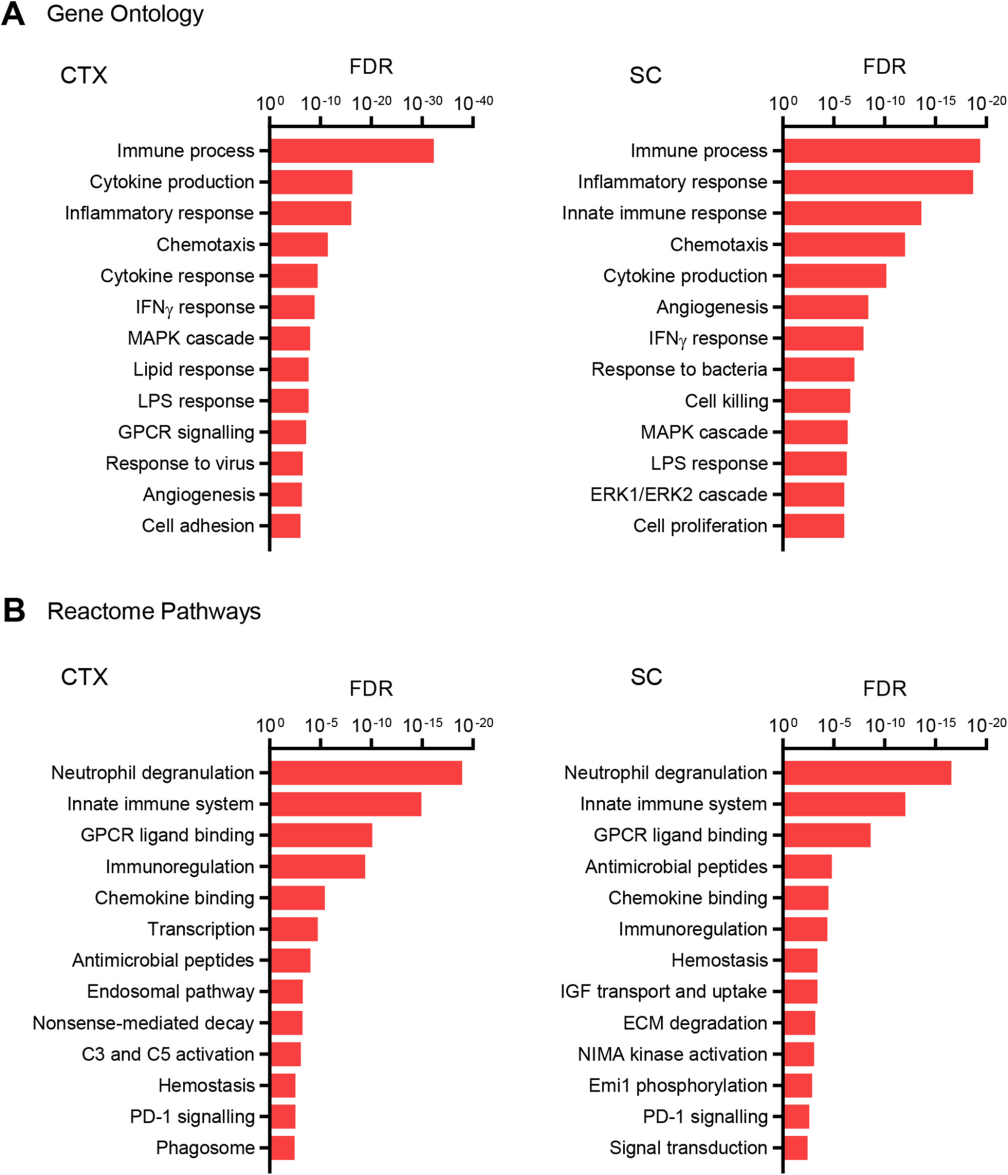
Gene ontology and molecular pathway analyses of transcriptomic changes in recovery-phase microglia. **A** Statistical significance of overrepresented gene ontology annotations (biological pathway subcategory) among genes showing significantly higher expression (log_2_ fold change > 1, Benjamini–Hochberg adjusted *P*-value < 0.05) in recovery-phase relative to control microglia in the cortex (left panel) or spinal cord (right panel). **B** Statistical significance of overrepresented Reactome pathway annotations among genes showing significantly higher expression (log_2_ fold change > 1, Benjamini–Hochberg adjusted *P*-value < 0.05) in recovery-phase relative to control microglia in the cortex (left panel) or spinal cord (right panel)

**Fig. 5:**
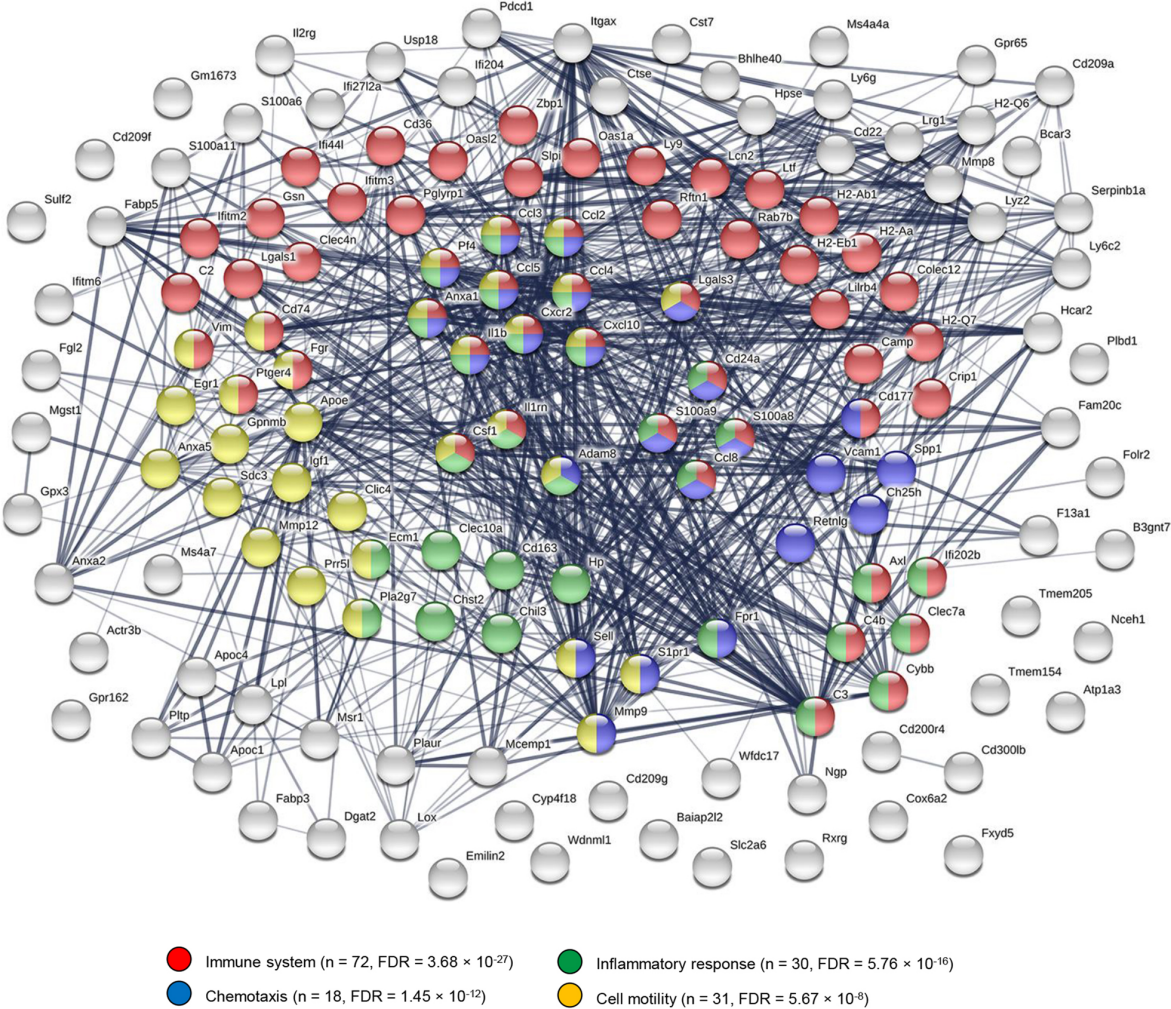
Protein–protein interaction network among genes showing increased expression in recovery-phase relative to control microglia isolated from the cortex. The network was generated from genes differentially expressed with LFC ≥ 2 and FDR < 0.05. The number of edges in the network (*n* = 780) was significantly greater than expected in a random gene sample of the same order (expected *n* = 142, p < 10^–16^). The network was enriched for proteins functioning in immune system processes (FDR = 3.68 × 10^–27^), inflammatory response (FDR = 5.76 × 10^–16^), chemotaxis (FDR = 1.45 × 10^–12^) and cell motility (FDR = 5.67 × 10^–8^), among other gene ontology annotations. The network was generated using STRING (https://string-db.org)

**Fig. 6:**
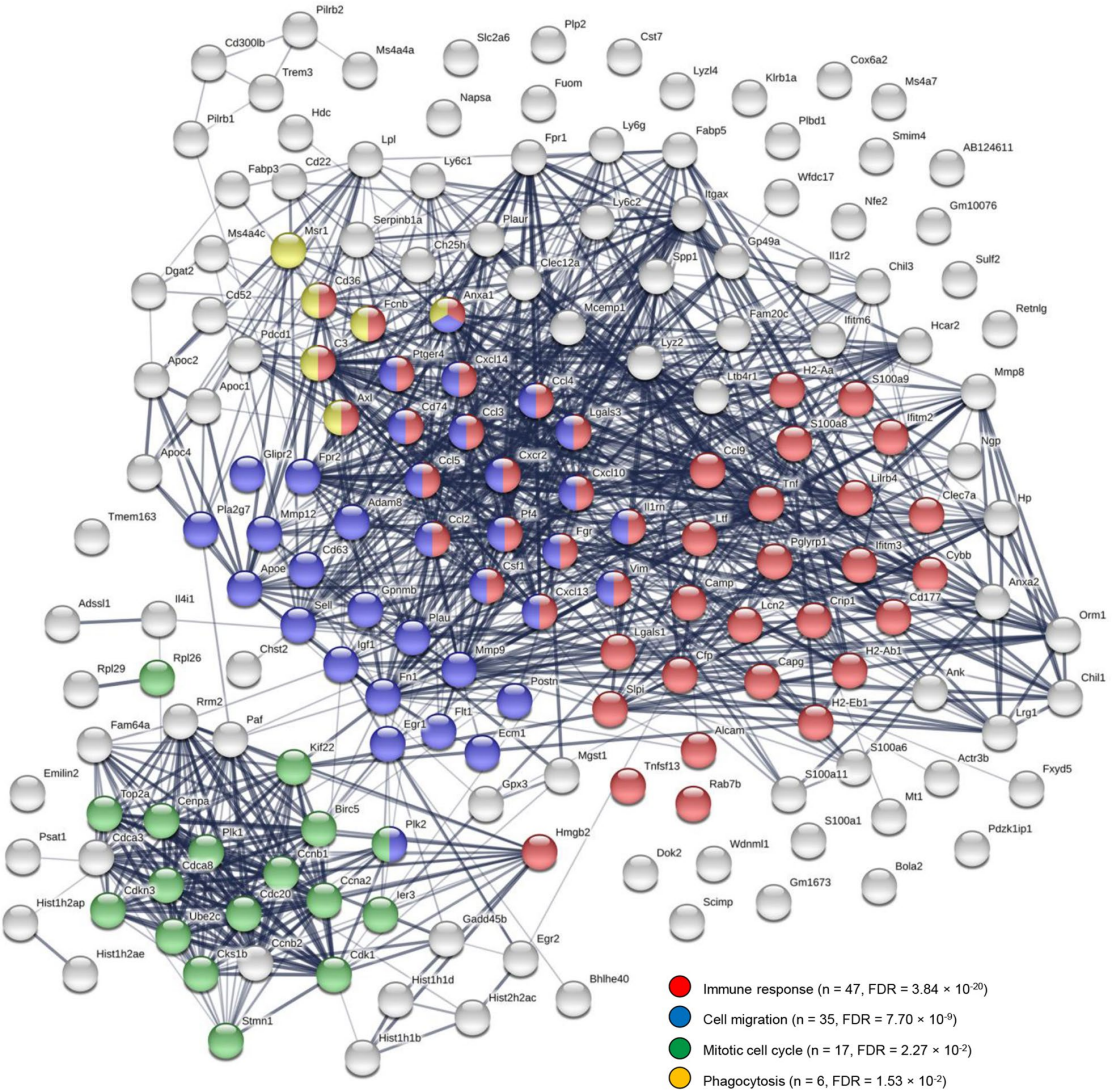
Protein–protein interaction network among genes showing increased expression in recovery-phase relative to control microglia isolated from the spinal cord. The network was generated from genes differentially expressed with LFC ≥ 1 and FDR < 0.05. The number of edges in the network (*n* = 1031) was significantly greater than expected in a random gene sample of the same order (expected *n* = 300, p < 10^–16^). The network was enriched for proteins functioning in immune response (FDR = 3.84 × 10^–20^), cell migration (FDR = 7.70 × 10^–9^), the mitotic cell cycle (FDR = 2.27 × 10^–2^), and phagocytosis (FDR = 1.53 × 10^–2^), among other gene ontology annotations. The network was generated using STRING (https://string-db.org)

Given the specific importance of microglia in the symptomatic resolution in rNLS8 mice, we investigated the enrichment of GO (Fig. 4A) and Reactome pathway (Fig. 4B) annotations among genes showing increased expression in recovery-phase relative to control microglia. This comparison sought to identify transcriptional features of recovery-phase microglia relative to those at resting state prior to hTDP-43 insult. Overrepresentation of GO and Reactome terms were consistent with activation of inflammatory and immunological processes, phagocytosis, microglial chemotaxis, ERK and chemokine signalling and responses to IFNγ, IFNα, IL-1, and TNF (Fig. 4A-B). Elucidating protein–protein interaction networks among factors showing increased expression in recovery-phase relative to control microglia in the cortex (Fig. 5) and spinal cord (Fig. 6) using the STRING database [19] in both cases identified highly interconnected networks dominated by genes involved in immunological and inflammatory response, cell migration and endocytic processes. While the non-equivalent signal–noise characteristics of the cortical and spinal cord differential expression contrasts precluded a direct comparison between these networks, the protein cluster relating to the mitotic cell cycle observed specifically in the spinal cord network was notable insofar as it comports with the greater microgliosis response observed during recovery in this anatomic site [14].

**Fig. 7:**
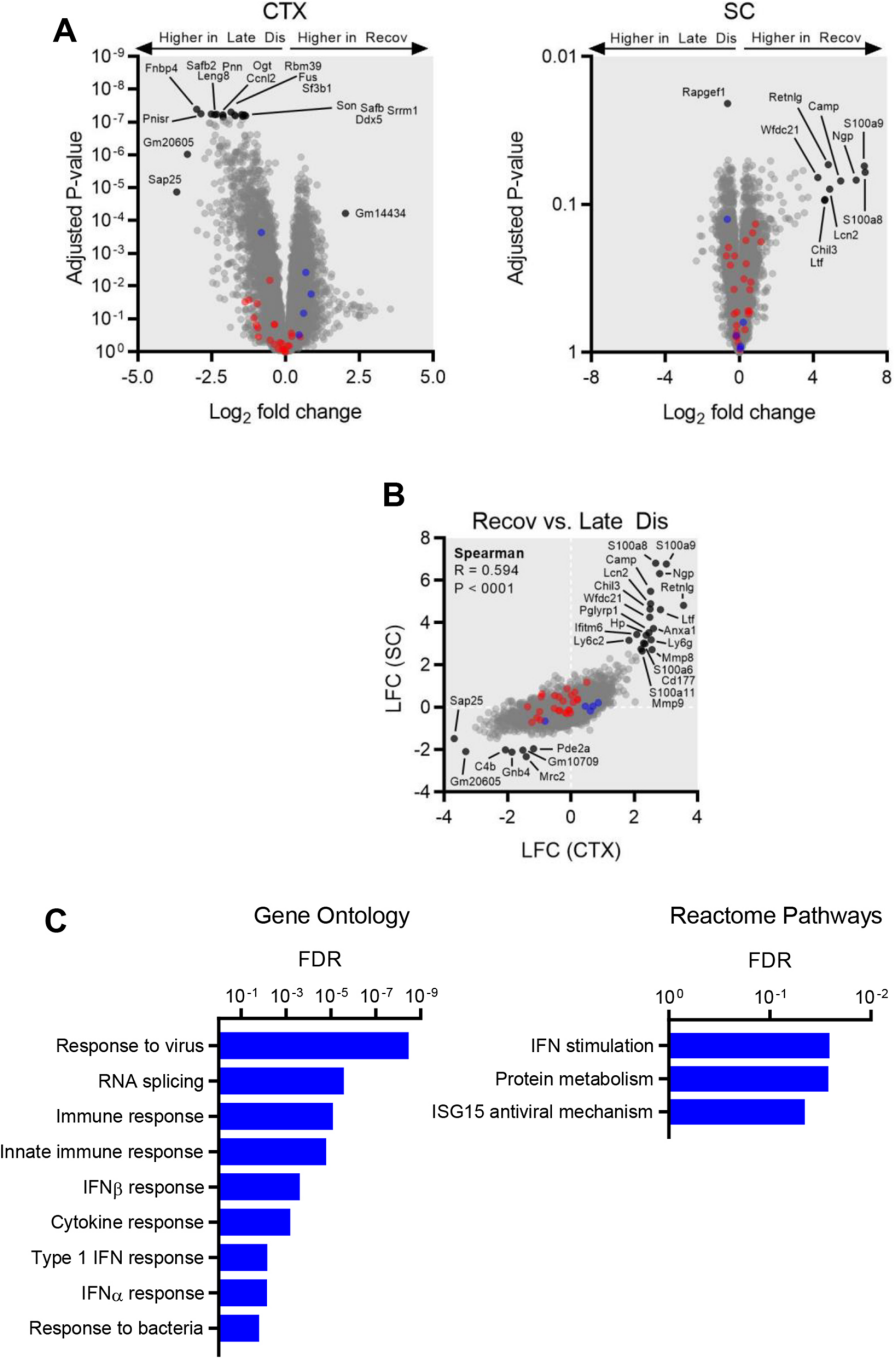
Gene expression differences between microglia isolated from rNLS8 mice in late disease vs. recovery phase. **A** Volcano plots illustrating the magnitude (as log_2_ fold change) and statistical significance (as Benjamini–Hochberg adjusted *P*-values) of changes in the expression of individual genes in microglia at recovery relative to late disease microglia isolated from the cortex (left panel) or spinal cord (right panel). Canonical DAM genes reported to be upregulated under neurodegenerative conditions are marked in red, whereas canonically downregulated DAM genes are marked in blue. **B** Scatter plot of log_2_ fold change values derived from the comparison of recovery and late disease microglia expression profiles in the cortex (horizontal axis) and spinal cord (vertical axis). Spearman correlation metrics are denoted as an assessment of the consistency of microglial gene expression changes across these two anatomical sites. Canonical DAM genes reported to be upregulated under neurodegenerative conditions are marked in red, whereas canonically downregulated DAM genes are marked in blue. (**C**) Statistical significance of overrepresented gene ontology annotations (biological pathway subcategory, left panel) and Reactome pathway annotations (right panel) among genes showing significantly lower expression (log_2_ fold change < -1, Benjamini–Hochberg adjusted *P*-value < 0.05) in recovery-phase relative to late-disease microglia in the cortex

As microglia undergo a recovery-promoting reaction specifically when the expression of hTDP-43 is suppressed (i.e., between the late disease and recovery phases in the present study) [14], we prosecuted differential expression analyses comparing recovery-phase to late disease microglia to isolate the gene expression changes occurring concurrently with the neuroprotective microglial switch (Fig. 7A). Given the variance in late disease spinal cord expression profiles already noted, we found limited power to resolve statistically significant expression differences in this anatomic site, with increased expression of a gene cluster comprised of *S100a8*, *S100a9*, *Ngp*, *Camp*, *Retnlg*, *Wfdc21*, *Lcn2*, *Chil3* and *Ltf*, and decreased expression of *Rapgef1* the most notable observations. In the cortex, however, while DAM genes were generally stable in expression between late disease and the recovery phase, many other genes (i.e., genes not part of the DAM signature) showed differential expression in the latter cohort, with the majority of these factors downregulated. As observed for spinal cord microglia, the gene cluster exemplified by calprotectin (*S100a8* and *S100a9*) was also upregulated in recovery-phase microglia from the cortex, albeit at a lesser magnitude (Fig. 7B). Genes that showed lower expression in recovery relative to late disease cortical microglia were enriched in GO and Reactome pathway annotations relating to immune response, particularly those downstream of type I interferons (Fig. 7C).

**Fig. 8:**
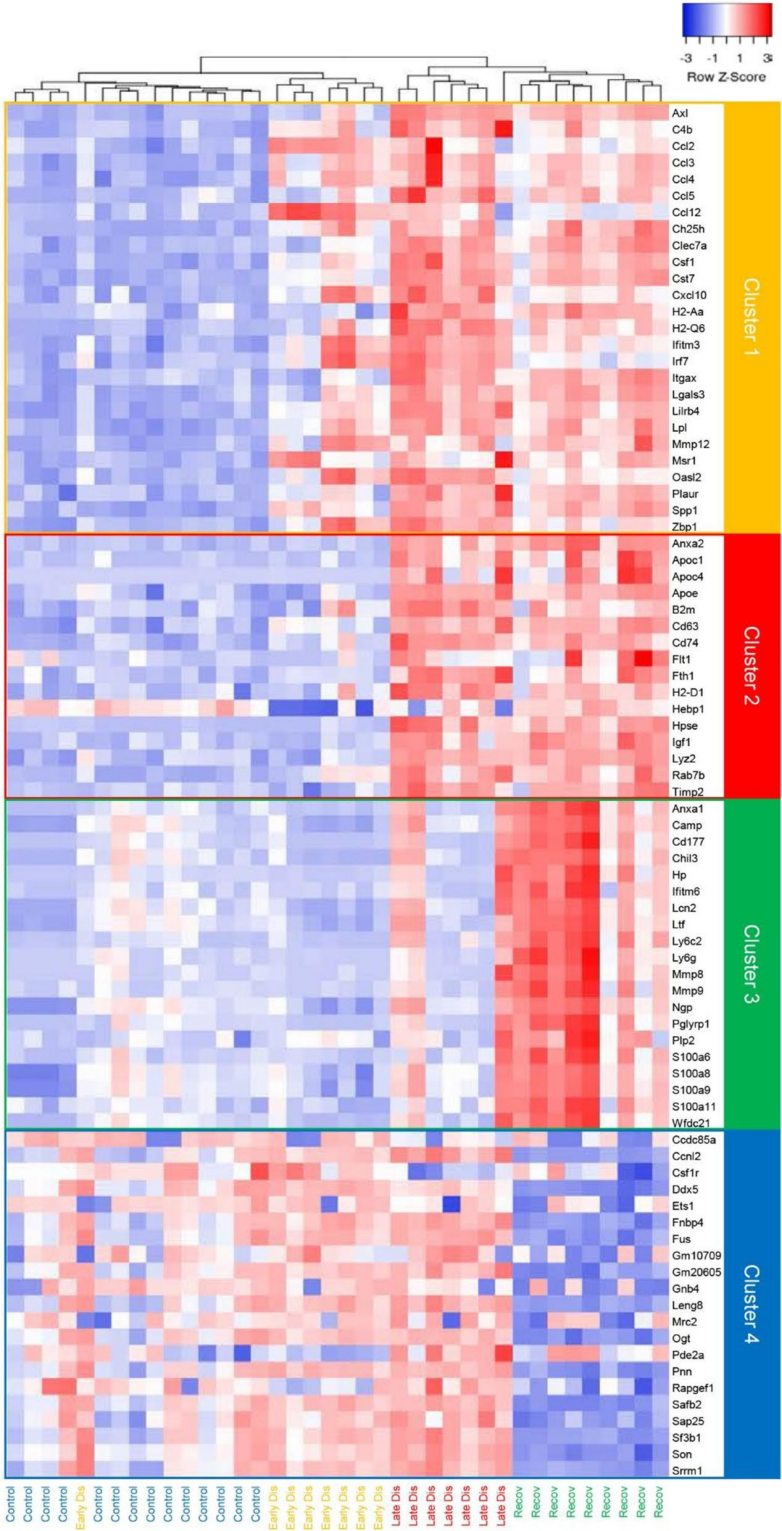
An expanded suite of disease- and recovery-associated genes in microglia isolated from the rNLS8 cortex. Unsupervised hierarchical clustering of expression values (normalized to row Z-scores) of an expanded suite of 89 disease- and recovery-associated genes in cortical microglia isolated from rNLS8 mice at baseline (control), early disease, late disease and recovery. Clustering used the ward.D method with Euclidean distance. Four discrete gene clusters are identified, where Cluster 1 comprises genes that show elevated expression in early disease relative to control that persists or is further elevated in late disease and recovery, Cluster 2 comprises genes that first show elevated expression in late disease that persists in recovery, Cluster 3 comprises genes with elevated expression specifically during recovery, and Cluster 4 comprises genes that show decreased expression specifically during recovery

Finally, we assembled a synthesis of the dynamic changes in the expression of a set of genes that showed highly altered expression in rNLS8 cortical (Fig. 8) or spinal cord microglia (Additional file 1: Figure S9) across disease stages. In the cortex, where expression profiles most clearly delineated the treatment cohorts, the gene set encompassed four clusters, where Cluster 1 defined genes with elevated expression in early disease that persisted through to recovery, Cluster 2 defined genes with elevated expression presenting first at late disease and persisting to recovery, Cluster 3 defined genes with low basal expression that was elevated specifically in recovery, and Cluster 4 defined genes with high basal expression that was suppressed specifically in recovery (Fig. 8). In the spinal cord, where significant gene expression changes were not observed at early disease nor from late disease to recovery, only Clusters 2 and 3 could be readily identified (Additional file 1: Figure S9).

## Discussion

### Microglial expression features at early disease

**Fig. 9:**
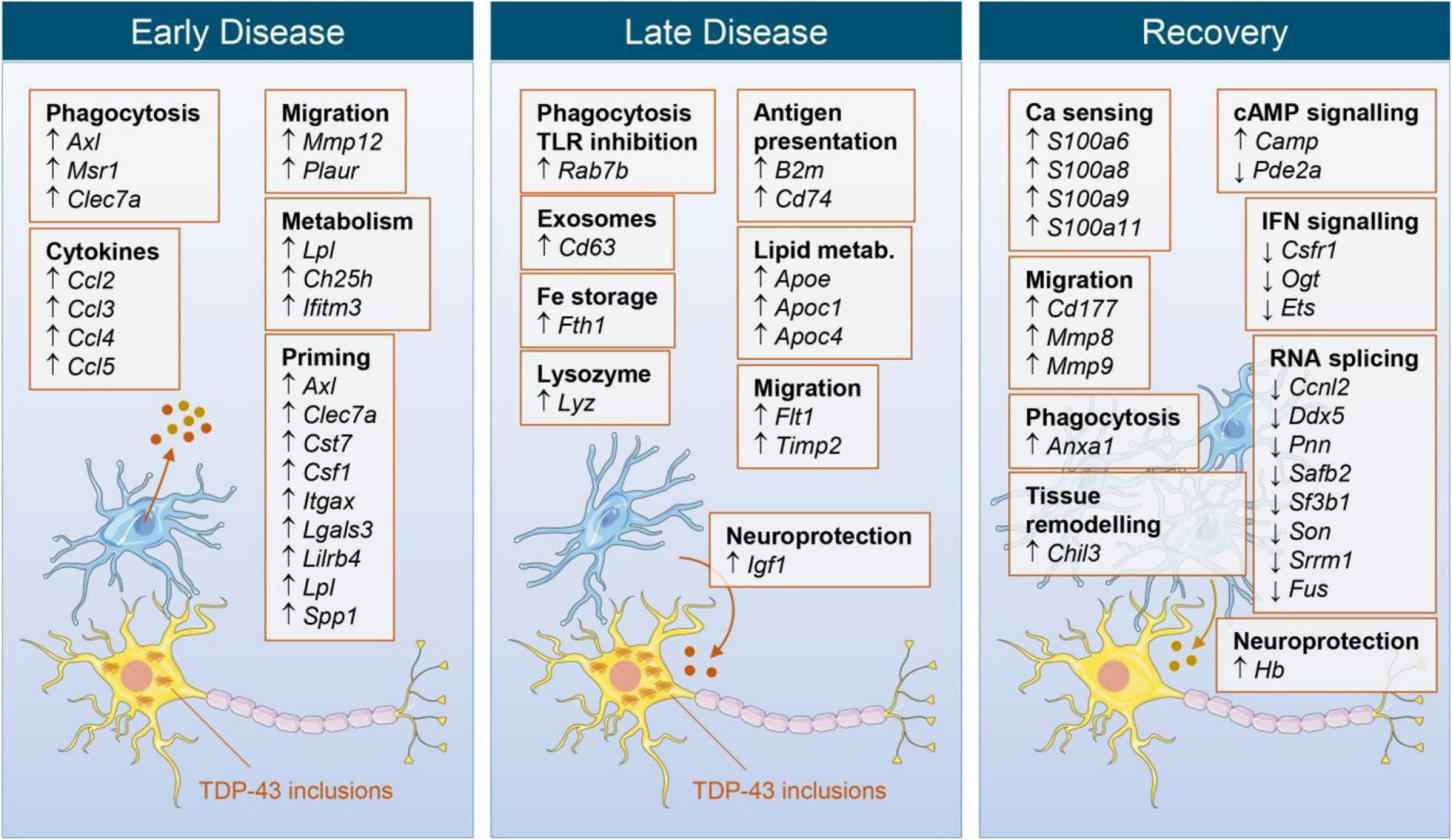
Abstracted schema of key microglial gene expression features at each stage of disease and recovery in rNLS8 mice

While the role of microglia in ALS remains contentious, a rational model, informed principally by functional studies of ALS risk genes, is that the native neuroprotective function of microglia is subverted in the context of pathological germline variants. However, approximately 90% of ALS presentations lack an overt familial etiology, with an accumulation of wild type TDP-43 aggregates understood to be the causal pathologic event. Thus, in sporadic ALS – as in the rNLS8 model – microglia may exert neuroprotective effects potentially amenable to therapeutic modulation given a sufficient understanding of the underlying molecular mechanisms. In this study, we identified temporal microglial expression signatures associated with TDP-43 proteinopathy in rNLS8 mice. Key transcriptional features observed at each disease stage are abstracted in Fig. 9. While the proximal triggers for the differential microglial reaction in rNLS8 mice remain unknown, it is likely that the suppression of hTDP43 and restoration of nuclear mTPD-43 alter the dynamics of the neuronal damage and thus the cellular milieu to which microglia are evolved to respond. Our observation of phenotypic differences in resting cortical and spinal cord despite the suppression of hTDP-43 in control animals is interesting albeit unsurprising given that analogous differences in the baseline transcriptional profiles of microglia have been reported in various brain regions [27–29].

Our observation of early activation of microglia in rNLS8 mice comports with clinical imaging studies in which use of glial-specific PET ligands has demonstrated microglial activation in early disease [30, 31]. Transcriptomic features observed at early disease were indicative of a specific reaction state of microglia that is distinct from late disease and recovery. This phenotype does not include downregulation of surveillance or homeostatic microglial key genes such as *P2ry12*, *Cx3cr1* and *Tmem119*. Gene expression changes at early disease were consistent with activation of NF-κB signalling, in which *Axl*, *C4b*, *Ccl2*, *Ccl3*, *Ccl4*, *Ccl5*, *Clec7a*, *Csf1*, *Cxcl10* and *Spp1* are all known mediators. Such an ‘alert’ or hypersensitive microglial phenotype has been observed in models of ageing and chronic activation (low-level inflammatory stimuli) as opposed to those reported following acute stimuli such as LPS [32]. In the absence of a single-cell RNAseq study in rNLS8 mice, it is unclear whether the concurrent expression of surveillance and pro-inflammatory genes is due to the coexistence of two (or more) distinct microglial subpopulations.

The TAM receptor Axl is a key regulator of microglial function, particularly phagocytic clearance of apoptotic neurons, plaques and debris during inflammation and neurodegeneration [26]. Interestingly, clinical-stage inhibitors of AXL have been developed for oncology indications [33] and this regulatory node is amenable to chemical biology manipulation for functional studies of microglia in rNLS8 mice. Macrophage scavenger receptor 1 (*Msr1*) is another phagocytic receptor with elevated expression in early disease [34]. The proteasome adjuvant protollin has been reported to augment microglial clearance of amyloid deposits in APP mice in part by upregulating Msr1 [35], begging the question of whether a similar approach may be successful for ALS. Upregulation of chemoattractant chemokines such as *Ccl2*, *Ccl3*, *Ccl4*, *Ccl5* and *Cxcl10* at this timepoint may indicate early recruitment of T cells and monocytes to sites of TDP-43 deposition. Expression of matrix metalloproteinase 12 (macrophage metalloelastase; *Mmp12*) and plasminogen activator, urokinase receptor (uPAR; *Plaur*) may reflect tissue remodelling processes associated with cell motility. Early elevated expression of interferon-induced transmembrane protein 3 (*Ifitm3*), lipoprotein lipase (*Lpl*) and cholesterol 25-hydroxylase (*Ch25h*), the enzyme responsible for the synthesis of 25-hydroxycholesterol from cholesterol, signals a rapid alteration in lipid metabolism within rNLS8 microglia that precedes altered expression of apolipoproteins at late disease. Another factor observed at early disease, *Irf7*, is a major regulator of type I IFN signalling.

### Microglial expression features at late disease

Late disease also corresponded to a distinct microglial transcriptomic state in our RNAseq dataset, including apical expression of *Apoe*, *B2m*, *Cd63*, *Fth1*, *Igf1*, *Lyz* and *Timp2*. A key feature of late disease microglia was upregulation of the apolipoproteins *Apoe*, *Apoc1* and *Apoc4*, suggesting altered lipid metabolism, which is a crucial factor for Alzheimer’s disease and reported to be associated to TREM2 signalling in microglia [36]. The function of elevated *Cd74* at late disease may indicate enhanced endosomal trafficking and modulation of inflammatory signalling [37] and would be consistent with our observed upregulation of ERK signalling [38]. Expression of vascular endothelial growth factor receptor 1 (*Flt1*) facilitates microglial chemotaxis to amyloid-β plaques in Alzheimer’s disease [39] and may perform equivalent functions in rNLS8 late disease. Another key expression feature observed at late disease was insulin-like growth factor 1 (*Igf1*). IGF1 is a neurotrophic factor with functions in neurodevelopment, myelination, synaptogenesis and neurogenesis [40, 41]. Microglia are major sources of IGF1 during disease and development, where signalling via IGF1R promotes neuronal survival [42, 43] while suppressing neurotoxic TNF-α and ROS production [44]. Our observation of elevated *Igf1* expression at late disease is particularly notable in light of these observations and is consistent with neuroprotective microglial Igf1 production in the SOD1^G93A^ ALS model [45]. Another transcript of note at late disease is *Rab7b*, which has been reported to enhance lysosomal degradation of TLR4 in macrophages [46]. *Timp2* expression – also observed here at late disease – has similarly been described to suppress microglial inflammation by inhibiting the production of nitric oxide, TNF-α, IL-1β and ROS while enhancing expression of IL-10 [47].

### Microglial expression features at recovery

We observed a discrete and novel set of differentially expressed genes that discriminated recovery-phase microglia both from control and from early or late disease. A key feature was upregulation of the calcium-binding heterodimer calprotectin (*S100a8* and *S100a9*), a marker of microglial activity that is elevated in schizophrenia [48], multiple sclerosis [49] and Alzheimer’s disease [50], where it may contribute to amyloidogenesis [51] and activate TLR4 signalling [52]. The additional calcium sensors *S100a6* and *S100a11* were also upregulated during recovery. The latter has been reported to suppress neuronal apoptosis in a model of ischemic stroke [53]. We also observed elevated expression of Annexin A1 (*Anxa1*) during recovery. This factor plays a role in resolving microglial inflammation whilst enhancing the phagocytic clearance of amyloid-β [54, 55]. Upregulation of *Lcn2* may similarly serve to limit inflammation and restore homeostasis in reactive microglia [56, 57]. Another upregulated neuroprotective factor was haptoglobin (*Hb*), which facilitates the microglial scavenging of neurotoxic haemoglobin [58]. Signalling through the cAMP-PKA-CREB axis is a key regulator of microglial activity. Our observed upregulation of the protein kinase A (PKA) subunit *Camp* and downregulation of the cAMP-hydrolyzing enzyme *Pde2a* may be indicative of enhanced signalling through this neuroprotective pathway. Consistent with this view, small molecule inhibitors of PDE2A showed efficacy in models of Alzheimer’s disease [59]. cAMP also enhances microglial filopodia formation [60], which, with concurrent *Cd177*, *Mmp8* and *Mmp9* expression, may signal ongoing glial migration at this timepoint.

The cohort of genes downregulated in recovery-phase microglia was characteristic of attenuation in interferon signalling, potentially reflecting a resolution of microglial responses upon clearance of TDP-43 aggregates [14]. This was exemplified by decreased expression of *Csf1r*, *Ogt* and the interferon-responsive transcription factor *Ets1*. Several genes involved in RNA splicing were downregulated during recovery including *Ccnl2*, *Ddx5*, *Pnn*, *Safb2*, *Sf3b1*, *Son*, *Srrm1* and *Fus*, the latter being an ALS risk gene [61, 62]. As discussed previously [14], while the function is unclear, this observation is notable given the role of TDP-43 in RNA metabolism. Of the 32 OMIM-recognized ALS risk genes that have known murine orthologs, we also noted mildly elevated expression of *Anxa11*, *Ccnf*, *Chchd10*, *Optn*, *Tardbp* and *Tuba4a* expression in cortical microglia in early or late disease, whereas *Pfn1* was mildly suppressed in late disease. None showed differential expression in spinal cord microglia. Of other strongly downregulated factors during recovery, *Fnbp4*, *Gnb4*, *Leng8*, *Rapgef1* and *Sap25* lack described functions in microglia and would be interesting subjects of future study. It is vital to note that while the expression signature observed here is concurrent with microglia-dependent recovery reported previously in rNLS8 mice [14], it remains unknown which transcriptional features are neuroprotective and which are secondary to the resolution of pathological processes following hTDP-43 suppression. Experimental studies to delineate the functionally important pathways are a priority for subsequent research.

## Conclusions

Several limitations of this study warrant mention. First, we observed significantly more expression variability in the spinal cord than in cortical isolates. It is unknown whether this reflects technical variability in experimentation, more microglial heterogeneity in the spinal cord or DOX biodistribution, although the fact that three outlying spinal cord specimens were processed together supports the former explanation.

Overall, our study identified novel transcriptomic features associated with microglial responses to TDP-43 proteinopathy. These were consistent with a neuroprotective function of microglia in rNLS8 mice, with increased transcript levels for key phagocytic regulators and chemokines at early disease. We observed a discrete set of differentially expressed genes at recovery, which included putative neuroprotective factors. Single-cell RNAseq studies will be required to assess whether the transcriptional phenotypes observed reflect dynamic changes in microglial subpopulations in rNLS8 mice, as was the case for DAM in 5XFAD and other disease models. If such subpopulations do exist in the rNLS8 model and moreover show discrete responses to TDP-43 pathology, this may account for ostensibly surprising features in the bulk transcriptional signatures, such as strong upregulation of proinflammatory calprotectin against a background of general resolution in the inflammatory expression signature seen during the recovery phase. Many of identified regulatory nodes identified in rNLS8, such as Axl, Pde2a, Flt1 and Csf1/Csf1r can be modulated using existing small and large molecules, while the use of stem cell or adeno-associated viral vectors may be of interest for delivering neuroprotective factors such as Igf1. Given the pathobiological convergence of ALS and other neurodegenerative diseases on the accumulation of protein aggregates, enhancing the phagocytic and protective functions of microglia whilst mitigating potentially toxic neuroinflammation may be an attractive new therapeutic modality across multiple neuroscience indications.

## Supplementary Information

**Additional file 1**. Supplemental figures and tables.

**Additional file 2**. Comparison of principal component scores between male and female rNLS8 control mice.

**Additional file 3**. Relative abundances of genes differentially expressed in microglia isolated from the spinal cord and cortex of rNLS8 control mice.

**Additional file 4**. Longitudinal differential expression “topologies” of genes in cortical and spinal cord microglia.

## Data Availability

The RNAseq data reported in this study are available in the NCBI Sequence Read Archive (Accession Number PRJNA624791).

## Abbreviations

ALS: Amyotrophic lateral sclerosis
CNS: Central nervous system
DAM: Disease-associated microglia
DOX: Doxycycline
GO: Gene ontology
hTDP-43: Human TDP-43
mTDP-43: Mouse TDP-43
NEFH: Neurofilament heavy chain
PCA: Principal component analysis
pTDP-43: Phosphorylated TDP-43
