# Supplementary material for: Microglial transcriptome analysis in the rNLS8 mouse model of TDP-43 proteinopathy reveals discrete expression profiles associated with neurodegenerative progression and recovery
Source: Acta Neuropathol Commun. 2021 Aug 19;9:140. doi: 10.1186/s40478-021-01239-x (PMC8377972; doi:10.1186/s40478-021-01239-x)
Supplement: Supplementary file 1 — Additional file 1. Supplemental figures and tables. [file 40478_2021_1239_MOESM1_ESM.docx]

Supplemental Materials for:

**Microglial transcriptome analysis in the rNLS8 mouse model of TDP-43 proteinopathy reveals discrete expression profiles associated with neurodegenerative progression and recovery**

Mandana Hunter^1^, Krista J. Spiller^1^, Myrna A. Dominique^1^, Hong Xu^1^, Francis W. Hunter^2^, Terry C. Fang^2^, Rebecca G. Canter^3^, Christopher J. Roberts^3^, Richard M. Ransohoff^4^, John Q. Trojanowski^1^, Virginia Man-Yee Lee^1*^

*^1^Center for Neurodegenerative Disease Research (CNDR), Institute on Aging, Department of Pathology and Laboratory Medicine, Perelman School of Medicine, University of Pennsylvania, Philadelphia, PA, USA;*

*^2^School of Medical Sciences, University of Auckland, New Zealand;*

*^3^Biogen, Cambridge, MA, USA;*

*^4^Third Rock Ventures, Boston, MA, USA.*

**Corresponding author email: vmylee@upenn.edu*

**Supplemental Data File**

| **File** | **Description** |
| --- | --- |
| 1 | Comparison of principal component scores arising from PCA of expression profiles of cortical or spinal cord microglia isolated female and male mice in the control cohort.  P-values were computed by Mann-Whitney test. |
| 2 | List of 231 differentially expressed genes (Benjamini-Hochberg <0.05; LFC >1) between microglia isolated from the cortex and spinal cord of control animals. |
| 3 | Longitudinal differential expression analysis in rNLS cortical and spinal cord microglia. |

**1. Supplemental Figures**


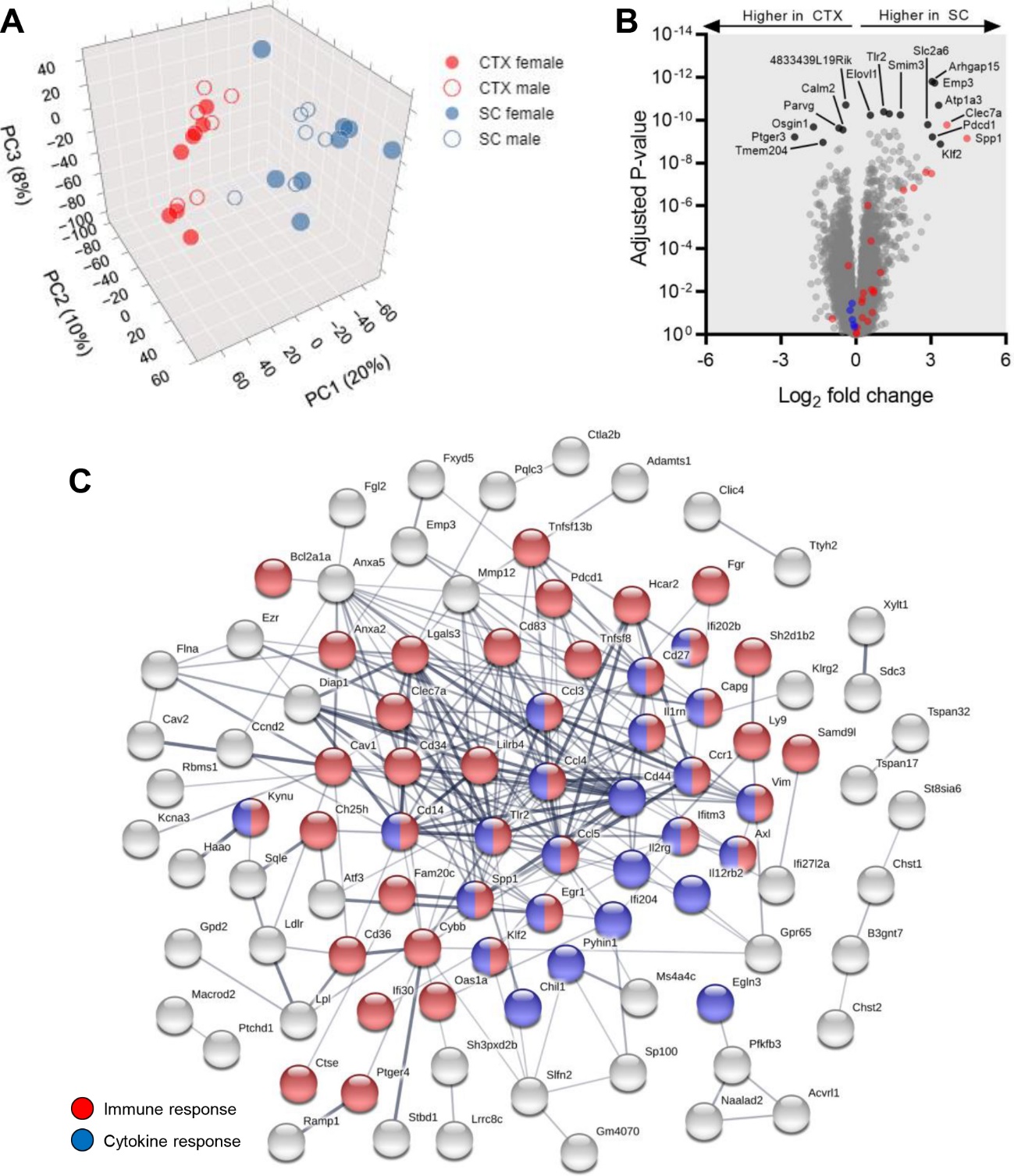


Supplemental Figure S1

**Supplemental Figure S1 (preceding page).** (a) Principal component analysis of transcriptomic data (as quantile-normalized, log_2_-transformed FPKM) obtained for microglia isolated from the cortex (left panel) or spinal cord (right panel) of male and female control mice. Scores for the first three principal components are represented in three-dimensional scatter plots, where data points correspond to microglial isolates from individual animals and the fraction of total variance explained by each principal component is denoted in axis labels. Principal component analysis used unit variance scaling and singular value decomposition with imputation. (b) Volcano plots illustrating the magnitude (as log_2_ fold change) and statistical significance (as Benjamini-Hochberg adjusted *P*-values) of changes in the expression of individual genes in control cortical relative to control spinal cord microglia. Canonical DAM genes reported to be upregulated under neurodegenerative conditions are marked in red, whereas canonically downregulated DAM genes are marked in blue. (c) Protein-protein interaction network among genes showing increased expression in control spinal cord relative to control cortical microglia (LFC ≥ 1; FDR < 0.01). The number of edges in the network (*n* = 250) was significantly greater than expected by random sampling (expected *n* = 73). The network was enriched for proteins function in immune system processes (FDR = 1.32 × 10^-7^) and cytokine response (FDR = 5.66 × 10^-3^). Network generated using STRING (https://string-db.org).


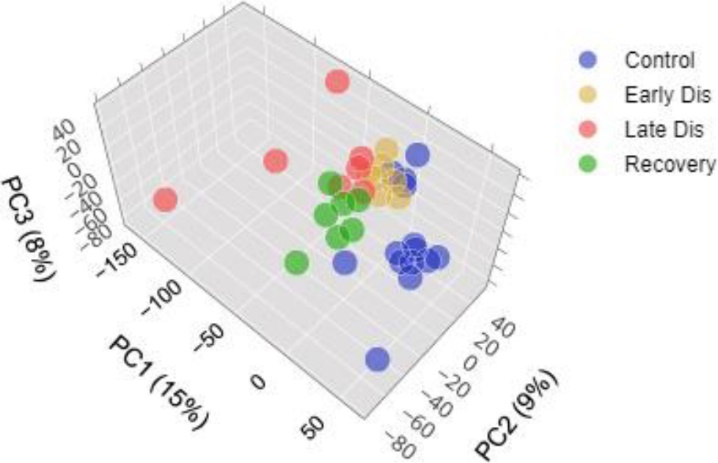


**Supplemental Figure S2.** Principal component analysis of transcriptomic data (as quantile-normalized, log_2_-transformed FPKM) obtained for microglia isolated from the spinal cord of control mice or animals in early disease, late disease and recovery phases, with two outlying recovery animals and one outlying early-disease animal excluded as identified in Figure 1C of the manuscript proper. Scores for the first three principal components are represented in three-dimensional scatter plots, where data points correspond to microglial isolates from individual animals and the fraction of total variance explained by each principal component is denoted in axis labels. Principal component analysis used unit variance scaling and singular value decomposition with imputation.


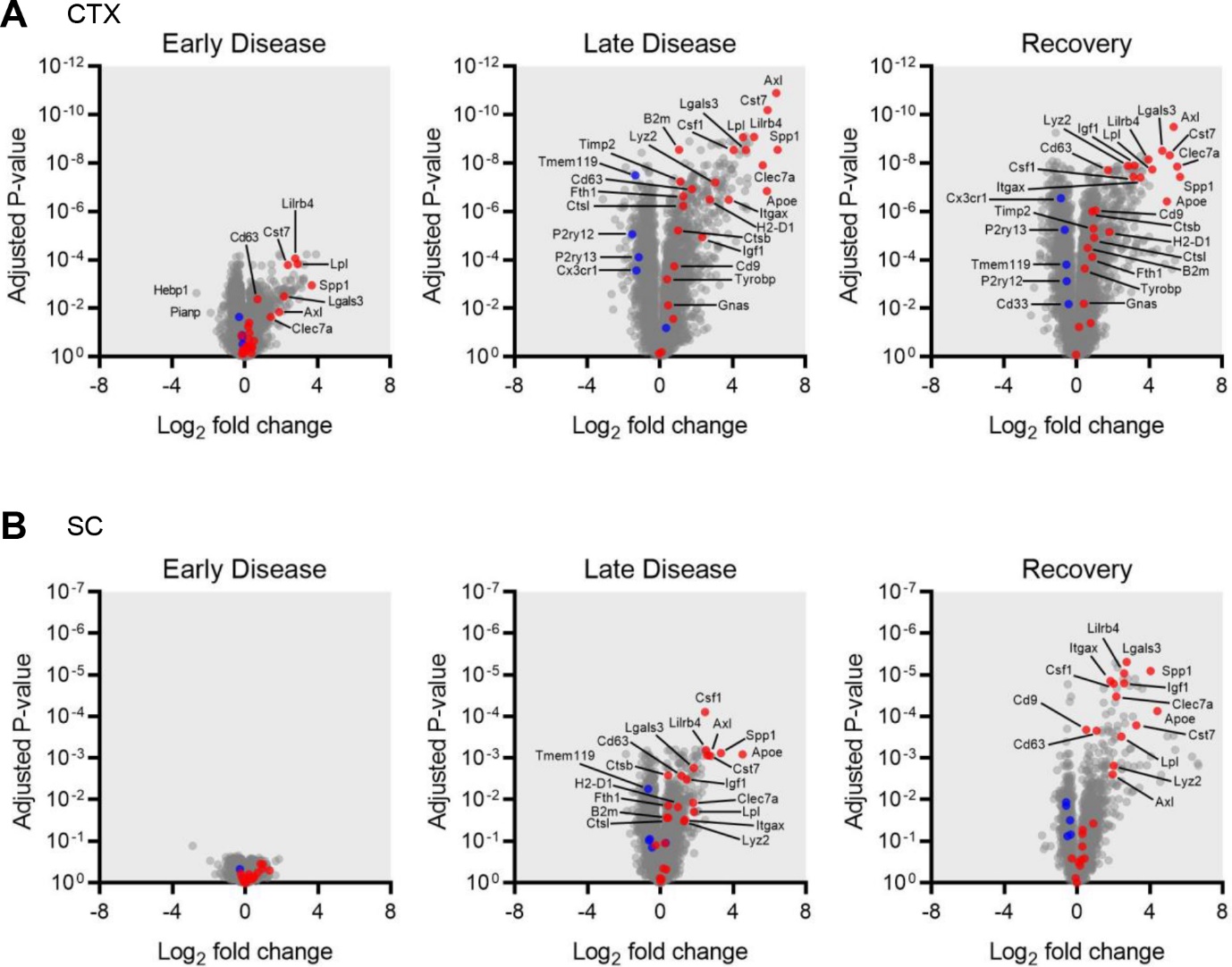


**Supplemental Figure S3.** Altered expression of disease-associated microglia (DAM) genes in microglia isolated from rNLS8 mice. (a) Volcano plots illustrating the magnitude (as log2 fold change) and statistical significance (as Benjamini-Hochberg adjusted P-values) of changes in the expression of individual genes in cortical microglia at early disease, late disease and recovery, in each case relative to cortical control microglia. Canonical DAM genes reported to be upregulated under neurodegenerative conditions are marked in red, whereas canonically downregulated DAM genes are marked in blue. (b) The equivalent analysis as (a) for microglia isolated from the spinal cord. (c) Unsupervised hierarchical clustering of microglia isolates from the cortex (left panel) or spinal cord (right panel) according to the expression values (normalized to row Z-scores as depicted in the heatmap scales) of canonical DAM genes. The reported direction of expression changes under neurodegenerative conditions is represented by the color coding of row labels. Two distinct sample clusters, a ‘non-DAM cluster’ containing all control and most early disease samples, and the ‘DAM cluster’ containing all recovery and most late disease isolates, are demarcated in the heatmaps. Clustering used the ward.D method with Euclidean distance.

**
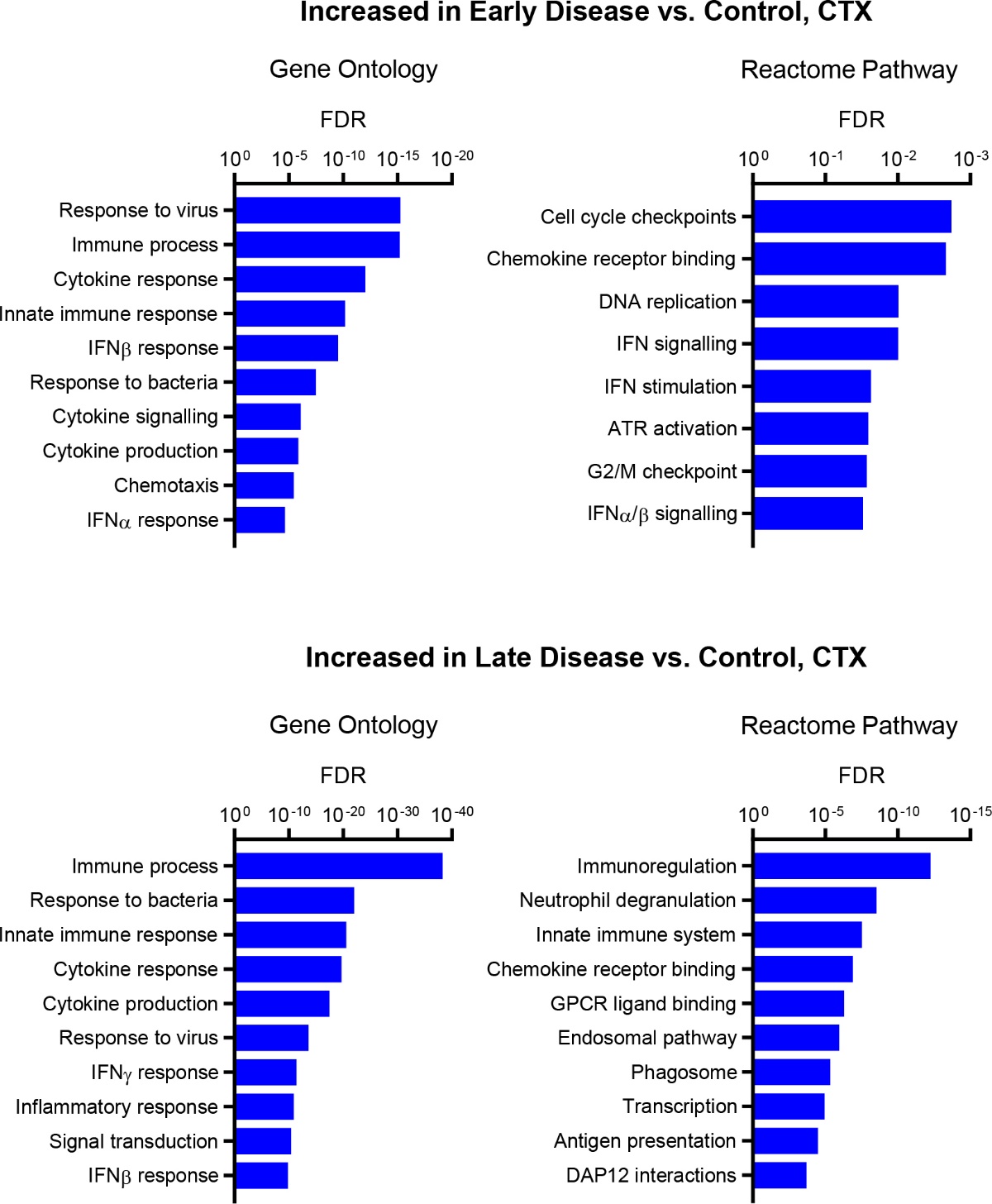
**

**Supplemental Figure S4.** Gene ontology and molecular pathway analyses of transcriptomic changes in early- and late-disease cortical microglia. Statistical significance of overrepresented gene ontology annotations (biological pathway subcategory) and KEGG pathway annotations among genes showing significantly higher expression (log_2_ fold change > 1, Benjamini-Hochberg adjusted P-value < 0.05) in early or late disease relative to control microglia in the cortex.


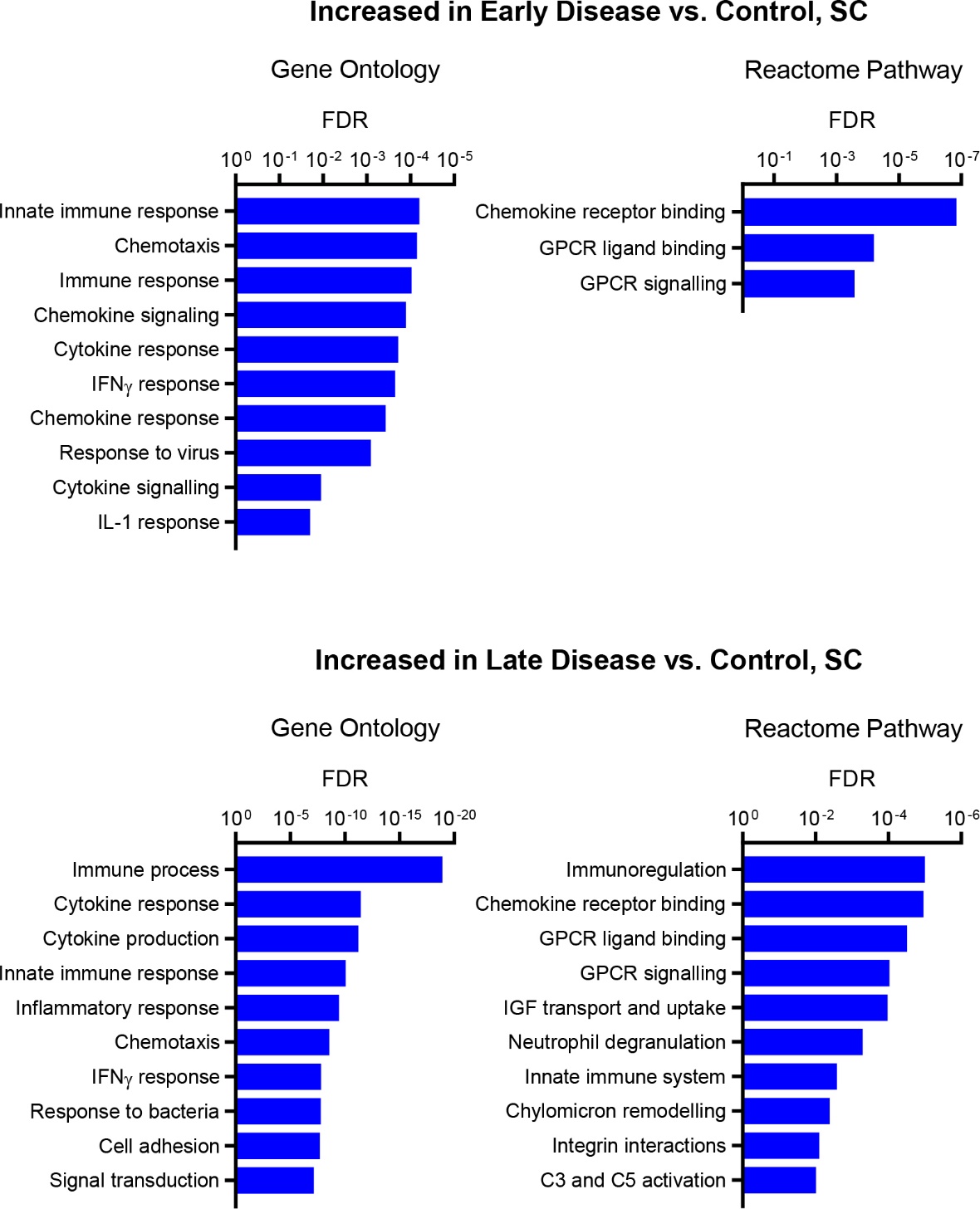


**Supplemental Figure S5.** Gene ontology and molecular pathway analyses of transcriptomic changes in early- and late-disease spinal cord microglia. Statistical significance of overrepresented gene ontology annotations (biological pathway subcategory) and KEGG pathway annotations among genes showing significantly higher expression (log_2_ fold change > 1, Benjamini-Hochberg adjusted P-value < 0.05) in early or late disease relative to control microglia in the spinal cord.


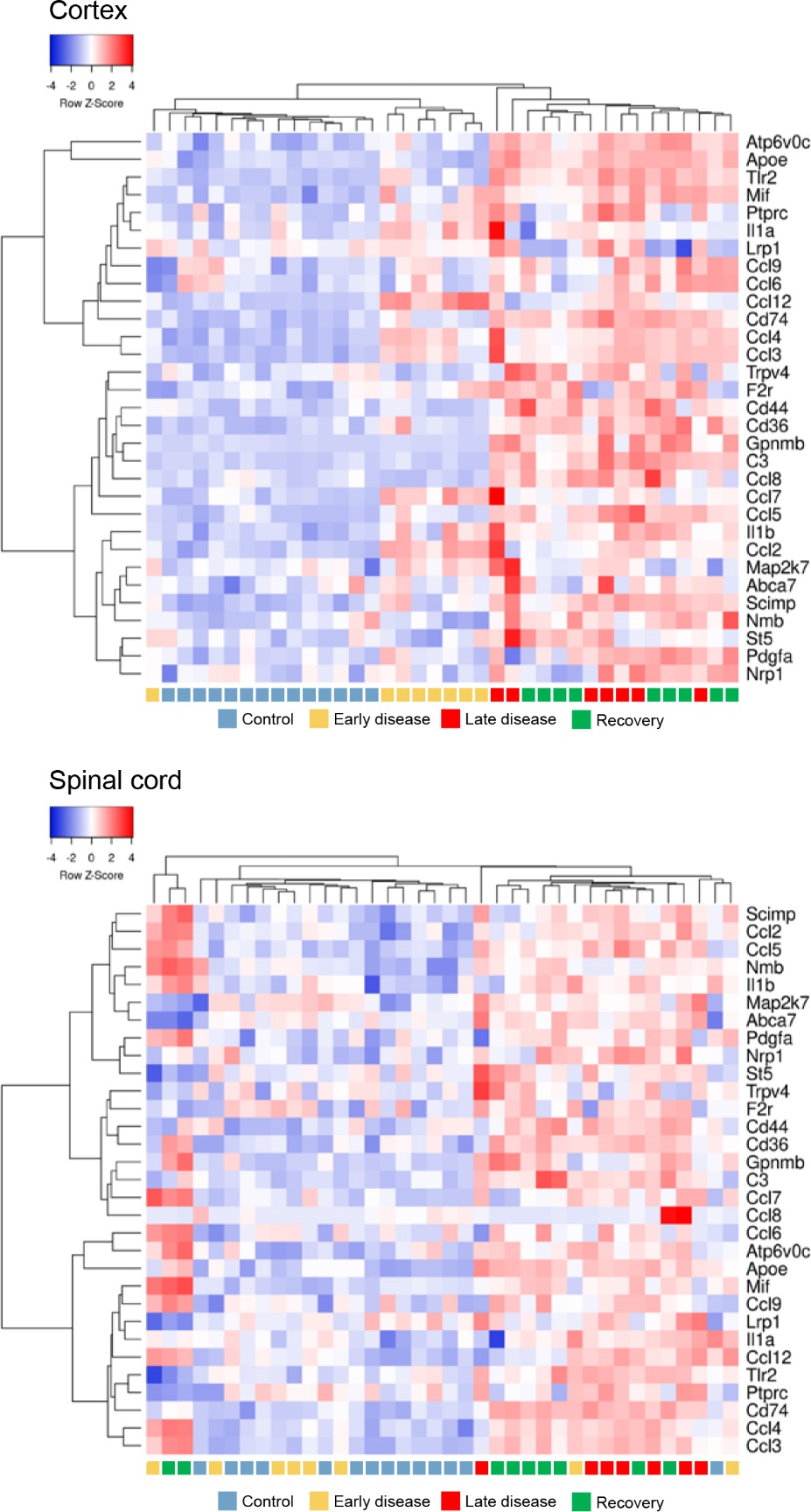


**Supplemental Figure S6.** Unsupervised hierarchical clustering of microglial isolates from the cortex and spinal cord based on expression Z-scores of genes involved in the ERK1 and ERK2 cascade (a subset of GO:0070372, ‘regulation of ERK1 and ERK2 cascade’). Clustering used the ward.D method with Euclidean distance.


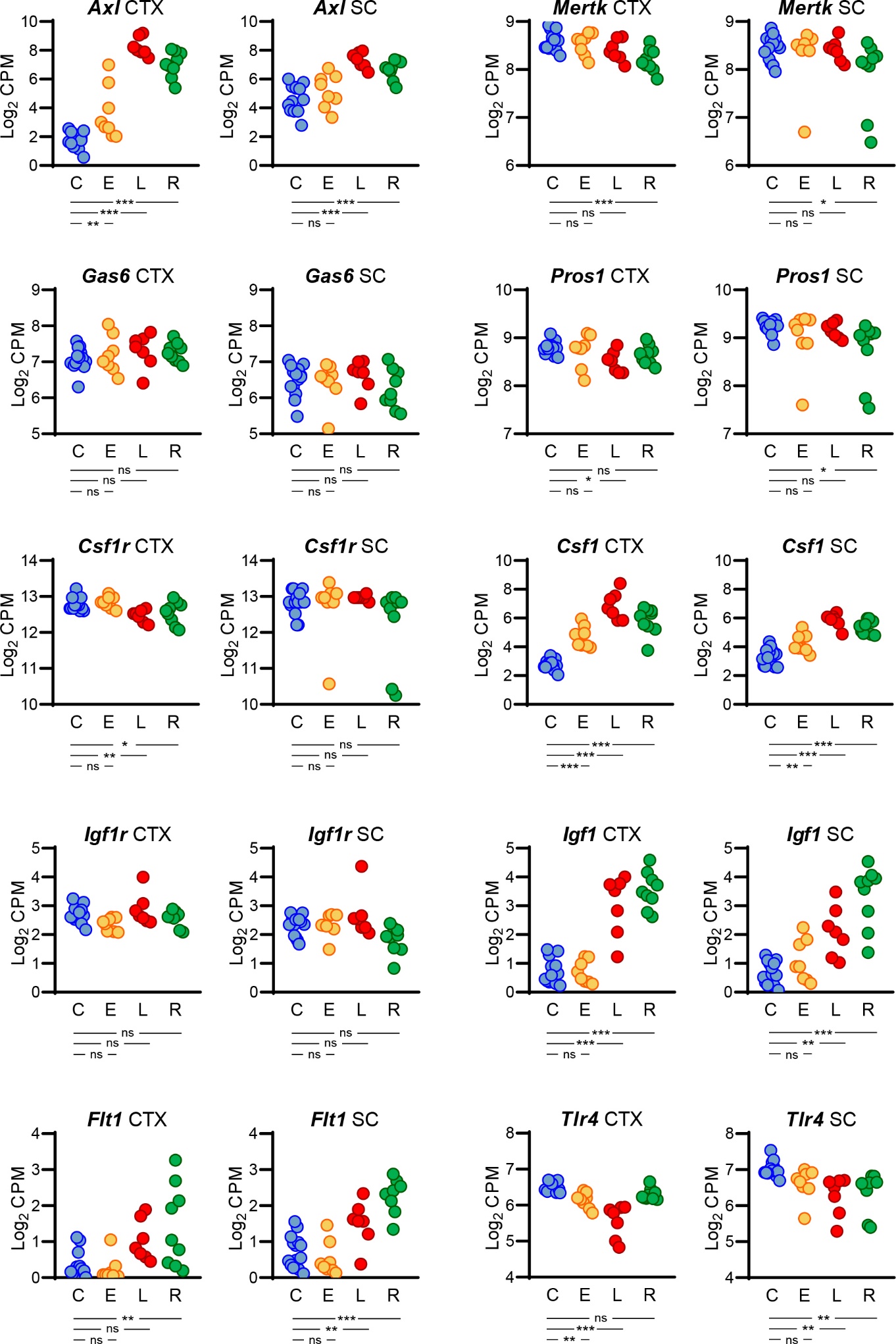


Supplemental Figure S7

**Supplemental Figure S7 (preceding page).** Expression values of key microglial receptors (*Axl*, *Mertk*, *Csf1r*, *Igf1r*, *Flt1*, *Tlr4*) and corresponding ligands (*Gas6*, *Pros1*, *Csf1*, *Igf1*) as a function of disease stage. Data are depth- and quantile-normalized log_2_ CPM. Statistical contrasts used one-way ANOVA with Dunnett’s multiple comparisons tests. ns = not significant, * = P < 0.05, ** = P < 0.01, *** = P < 0.001. C = control, E = early disease, L = late disease, R = recovery.


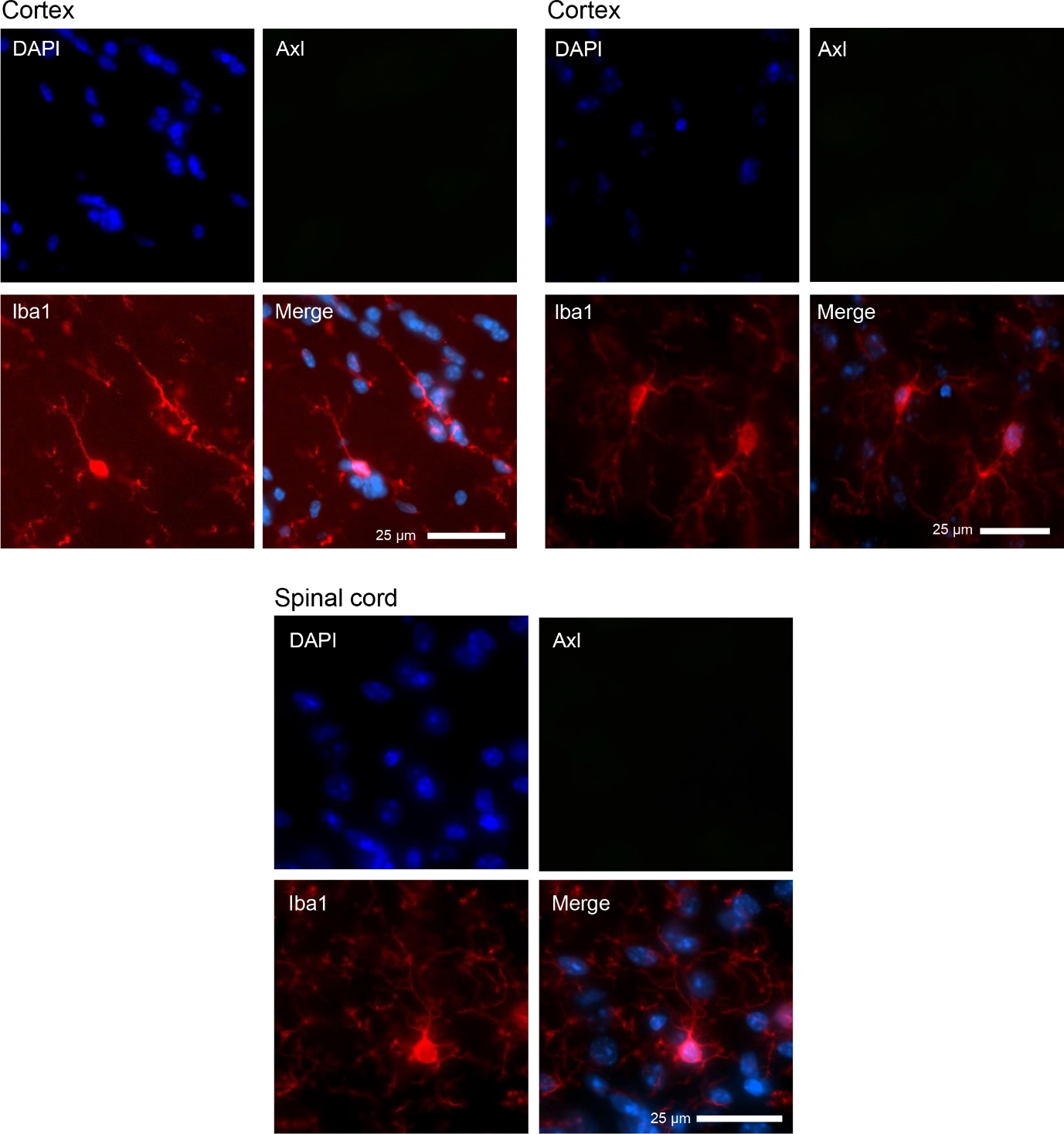


**Supplemental Figure S8.** Immunofluorescence staining for Axl and Iba1 in cortical and spinal cord sections from wild type (non-transgenic) littermates that were off dietary doxycycline for a period of 6 weeks (i.e., corresponding to the rNLS8 late disease timepoint shown in Fig. 3 of the main text).


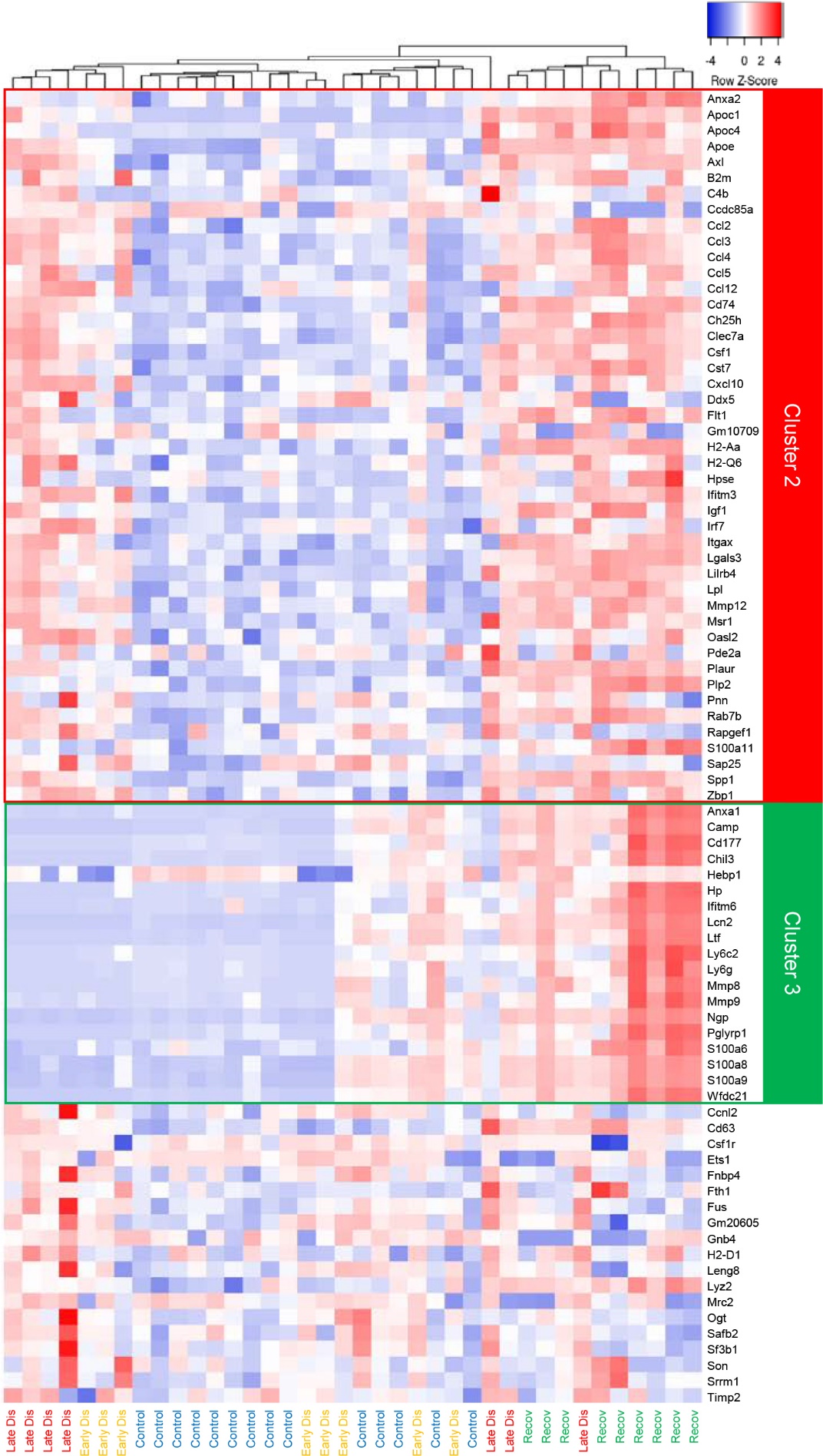


Supplemental Figure S9

**Supplemental Figure S9 (preceding page).** An expanded suite of disease- and recovery-associated genes in microglia isolated from the rNLS8 spinal cord. Unsupervised hierarchical clustering of expression values (normalized to row Z-scores) of an expanded suite of 89 disease- and recovery-associated genes in spinal cord microglia isolated from rNLS8 mice at baseline (control), early disease, late disease and recovery. Clustering used the ward.D method with Euclidean distance. Two discrete gene clusters are identified that correspond to those noted in Figure 8, where Cluster 2 comprises genes that first show elevated expression in late disease that persists in recovery, and Cluster 3 comprises genes with elevated expression specifically during recovery.

**2. Supplemental Tables**

**Table S1.** List of canonical DAM genes.

| **Gene** | **Protein name** | **Expression in DAM** |
| --- | --- | --- |
| *Apoe* | Apolipoprotein E | Increased |
| *Axl* | Tyrosine-protein kinase receptor UFO | Increased |
| *B2m* | Beta-2-microglobulin | Increased |
| *C1qb* | Complement C1q subcomponent subunit B | Decreased |
| *Ccl6* | C-C motif chemokine 6 | Increased |
| *Cd63* | CD63 antigen | Increased |
| *Cd9* | CD9 antigen | Increased |
| *Clec7a* | C-type lectin domain family 7 member A | Increased |
| *Csf1* | Macrophage colony-stimulating factor 1 receptor | Increased |
| *Cst7* | Cystatin F | Increased |
| *Cts3* | Cathepsin 3 | Decreased |
| *Ctsb* | Cathepsin B | Increased |
| *Ctsd* | Cathepsin D | Increased |
| *Ctsl* | Cathepsin L1 | Increased |
| *Fth1* | Ferritin heavy chain | Increased |
| *Gnas* | GNAS Complex locus | Increased |
| *H2-D1* | H-2 class I histocompatibility antigen, D-B alpha chain | Increased |
| *Hexb* | Beta-hexosaminidase subunit beta | Decreased |
| *Igf1* | Insulin-like growth factor I | Increased |
| *Itgax* | Integrin alpha-X | Increased |
| *Lgals3* | Galectin-3 | Increased |
| *Lilrb4* | Leukocyte immunoglobulin-like receptor subfamily B member 4 | Increased |
| *Lpl* | Lipoprotein lipase | Increased |
| *Lyz2* | Lysozyme C-2 | Increased |
| *Spp1* | Osteopontin | Increased |
| *Timp2* | Metalloproteinase inhibitor 2 | Increased |
| *Trem2* | Triggering receptor expressed on myeloid cells 2 | Increased |
| *Tyrobp* | TYRO protein tyrosine kinase-binding protein | Increased |
